# Symptom Trajectories and Clinical Subtypes in Post–COVID-19 Condition: Systematic Review and Clustering Analysis

**DOI:** 10.2196/72221

**Published:** 2025-07-18

**Authors:** Mingzhi Hu, Tian Song, Zhaoyuan Gong, Qianzi Che, Jing Guo, Lin Chen, Haili Zhang, Huizhen Li, Ning Liang, Guozhen Zhao, Yanping Wang, Nannan Shi, Bin Liu

**Affiliations:** 1 Institute of Basic Research in Clinical Medicine, China Academy of Chinese Medical Sciences, No 16, Nanxiao Street, Dongzhimen, Dongcheng District, Beijing, 100700, China, 86 18515189525

**Keywords:** post–COVID-19 condition, temporal dynamic, symptoms, subtypes, systematic review

## Abstract

**Background:**

Post–COVID-19 condition presents complex symptomatology involving multifaceted interactions, which has resulted in a current lack of comprehensive understanding of its disease trajectory. This knowledge gap significantly compromises the efficiency of symptom management and adversely affects patients’ quality of life.

**Objective:**

This study aims to comprehensively characterize the temporal evolution of post–COVID-19 condition by identifying core symptom clusters and clinical phenotypes, thereby enhancing understanding of the disease trajectory.

**Methods:**

The PubMed, Web of Science, and Embase databases were searched from December 1, 2019, to March 1, 2024. Observational studies related to the prevalence of symptoms in post–COVID-19 condition had been included. We conducted a meta-analysis to synthesize symptom prevalence across different follow-up intervals following PRISMA (Preferred Reporting Items for Systematic Reviews and Meta-Analyses) guidelines and used a network to explore interrelationships and co-occurrence patterns among symptoms, enabling the identification of core symptoms and changes over time. Clustering analysis was used to classify included studies into distinct clinical subtypes.

**Results:**

This study analyzed 155 sets of macrolevel data from 108 clinical studies, encompassing 63,771 patients. Fatigue was the most prevalent symptom across all 4 follow-up points (52%, 48%, 46%, and 54%). Dyspnea peaked at the third and sixth follow-ups (36% and 31%) and then declined steadily (28% and 22%). Subgroup analysis revealed that Africa reported the fewest symptoms overall, yet showed high early incidences of fatigue (68%, 95% CI 50%‐85%) and dyspnea (56%, 95% CI 15%‐98%). The Americas placed greater emphasis on symptom evolution within the first postinfection year, with notably higher prevalence of anxiety (60%, 95% CI 54%‐66%) and depression (36%, 95% CI 16%‐55%). Asia and Europe documented the most comprehensive symptom profiles, with Asia reporting lower early dyspnea rates (29%, 95% CI 18%‐40%) and Europe exhibiting more complex multisystem involvement during long-term follow-up. Network analysis showed that core post–COVID-19 symptoms evolved from early respiratory-neurological manifestations to chronic multisystem symptoms dominated by dizziness. Clustering analysis further indicated a progressive convergence of 2 initially distinct post–COVID-19 subtypes, with the acute inflammatory type becoming less prominent and gradually transitioning into a more chronic, persistent pattern.

**Conclusions:**

This study provides a comprehensive characterization of the dynamic evolution of post–COVID-19 condition symptoms and clinical subtypes, highlighting their multisystem involvement. The results reveal a progressive decline in respiratory symptoms over time, while neurological manifestations emerge as the most persistent and systemically impactful core symptoms. Our findings emphasize the need for region-specific surveillance and early warning systems informed by symptom progression patterns. By continuously monitoring the trajectories of symptom clusters, this approach offers valuable insights for identifying early warning signals and targeted intervention points in the management of postinfectious sequelae arising from future large-scale epidemics.

## Introduction

Studies have shown that approximately 10%‐30% of patients continue to experience persistent symptoms after nucleic acid test results turn negative or after discharge from the hospital, significantly impacting their daily lives [1-3]. Furthermore, some patients may experience symptoms lasting more than 2 years [4,5]. The World Health Organization defines the persistent symptoms following the acute phase of COVID-19 as “post–COVID-19 condition,” characterized by symptoms that persist or emerge at least 3 months after the initial SARS-CoV-2 infection and cannot be explained by other causes. As of March 2025, a total of 777 million people worldwide have been infected with COVID-19 [6]. Assuming a conservative estimate that 10% of recovered individuals will experience persistent symptoms, over 70 million people may be affected by the long-term sequelae of COVID-19. Post–COVID-19 condition presents with complex symptoms affecting multiple systems and organs throughout the body, with studies reporting over 200 types of symptoms [7]. Common symptoms include fatigue, postexertional malaise, cognitive impairment (brain fog), dyspnea, chest pain, as well as anxiety and depression [8,9]. A single patient may simultaneously exhibit symptoms across multiple systems, with these symptoms interacting and forming a vicious cycle. The heterogeneity and dynamic nature of these symptoms pose significant challenges to the treatment of post–COVID-19 condition. Over the past few years, numerous researchers have conducted studies to understand the pathophysiology, risk factors, and optimal management strategies for post–COVID-19 condition [10-12]. However, no definitive pharmacological treatments or interventions have been established to date, and current approaches primarily rely on comprehensive management or symptomatic relief. Against this backdrop of symptom-based care, existing studies have reported the prevalence of post–COVID-19 condition symptoms and proposed clinical subtypes, such as fatigue-dominant and musculoskeletal-dominant phenotypes. Nevertheless, most of these studies have relied on cross-sectional assessments, failing to capture the dynamic symptom patterns or phenotypic transitions over time, and have not prioritized the identification of core symptoms [13,14].

Since the concept of symptom networks was first introduced by Fried et al [15], this research paradigm has been widely applied in fields such as psychiatry, chronic disease management, and long-term oncology follow-up [16,17]. Symptom networks not only provide direct measurements such as symptom prevalence and severity but also uncover the complex interactions among symptoms through a range of network metrics, including node centrality, predictability, clustering, network connectivity, and transitivity. Core symptoms, identified via centrality and related indicators, often exert a disproportionate influence over the entire network. Targeting these symptoms may yield a “leverage effect,” leading to broader improvements in symptom management. Clustering analyses can detect co-occurring symptom patterns, pointing toward potential symptom groups and shared pathophysiological mechanisms [18]. They provide a theoretical and methodological foundation for exploring underlying mechanisms, refining symptom phenotyping, and identifying key therapeutic targets. Constructing dynamic symptom networks, in particular, allows for the identification of changes in intersymptom relationships over time [19]. This approach can help uncover patterns of symptom co-occurrence and mutual influence, contributing to the early detection and diagnosis of complex conditions such as post–COVID-19 condition. By identifying core and bridge symptoms and delivering interventions that specifically target these pivotal nodes, it becomes possible to disrupt symptom interaction pathways, rendering associated symptoms less connected and reducing their impact. This network-based strategy facilitates a shift from the traditional model of treating multiple symptoms independently to a more precise approach that targets the most influential symptom nodes [20], thereby enhancing symptom control and improving quality of life.

Although systematic reviews have investigated the incidence of post–COVID-19 condition symptoms, their inclusion of primary studies was limited to observation periods of up to 1 year at most, thereby precluding assessment of longer-term symptom trajectories [21]. These reviews also failed to explore the dynamic symptom patterns or phenotypic transitions of post–COVID-19 condition over time. Furthermore, the lack of timely updates in these studies has left symptom evolution patterns over the past 3 years largely unexplored. Notably, none of these systematic reviews have specifically examined the dynamic changes in core symptoms and symptom clusters as their primary research focus. Building upon this foundation, this study seeks to conduct a systematic evaluation and dynamic analysis of symptom data derived from published clinical investigations, with the primary objectives of identifying core symptom clusters associated with post–COVID-19 condition, elucidating their characteristic phenotypic evolution patterns, addressing the critical knowledge gap concerning long-term symptom trajectories extending beyond 1 year postinfection, and generating updated epidemiological evidence regarding symptom prevalence rates in this emerging clinical entity. The findings will contribute to providing a scientific basis for public health surveillance and stratified management of post–COVID-19 condition, thereby aiding in the optimization of medical resource allocation and the formulation of rehabilitation policies.

## Methods

### Study Design

This study was designed as a systematic review and meta-analysis of observational studies investigating post–COVID-19 condition combined with network analysis and clustering to identify longitudinal symptom patterns and clinical subtypes. We conducted a comprehensive literature search of published clinical studies from December 2019 to March 2025, extracting symptom prevalence data across different follow-up intervals (3rd, 6th, 12th, and 24th month). This temporal stratification allowed for the examination of symptom dynamics over time. To standardize the classification of reported symptoms across studies, we categorized each symptom into 1 of 9 system domains, including respiratory, nervous, circulatory, digestive, musculoskeletal, endocrine, skin, urogenital, and other. The classification framework was primarily based on the *International Classification of Diseases, 11th Revision* (*ICD-11*). For symptoms that could plausibly be assigned to multiple systems, such as fatigue, we determined the most appropriate category based on predominant pathophysiological relevance, as indicated by current literature. For example, fatigue was classified under neurological manifestations due to its established association with autonomic dysfunction and neuroinflammatory processes in post–COVID-19 condition. A complete list of symptoms and their corresponding system classifications is provided in Table S1 in Multimedia Appendix 1 to facilitate the reproducibility of the categorization process. The meta-analysis was used to estimate pooled symptom prevalence rates at each time point, enhancing comparability across studies. To further explore the interrelationships among symptoms and their evolution, we applied network analysis, which revealed the co-occurrence structures and central symptoms within each follow-up period. Subsequently, we performed clustering analysis to group population-level data into distinct clinical phenotypes based on symptom profiles, enabling the identification of population-level subgroups with similar post–COVID-19 condition presentations. This stepwise, multimethod analytical framework was designed to address the complexity and heterogeneity of post–COVID-19 condition symptoms and provide a comprehensive understanding of their temporal and structural patterns. We have registered this meta-analysis on PROSPERO (CRD42024537825). We followed the PRISMA (Preferred Reporting Items for Systematic Reviews and Meta-Analyses) statements for reporting our systematic review (Checklist 1) [22].

### Search Strategy

We searched the following databases from December 1, 2019, to March 1, 2025 (with the language restricted to English): PubMed, Embase, and Web of Science. MeSH (Medical Subject Headings) terms were combined with text words in the literature retrieval related to “post–COVID-19 condition,” “symptom,” and “follow-up”; for specific details on the search strategy, please refer to Multimedia Appendix 2. To find additional studies, we screened the relevant reference lists of the included studies and previous literature reviews including systematic reviews and meta-analyses. All search results were imported to an EndNote (version X9; Clarivate Analytics) tool to facilitate the screening of titles and abstracts.

### Data Collection and Extraction

Two independent reviewers (HL and GZ) worked together to screen eligible studies. The complete eligibility criteria are presented in Textbox 1. In the initial screening, they read titles and abstracts to include studies containing 1 or more intervention models. All potentially relevant studies were retrieved for full-text screening. A full-text review of potentially eligible studies then was conducted, and the reasons for exclusion in the full-text screening were recorded in the EndNote database. After screening, data were extracted independently by the reviewers using a data extraction form (LC and NL). The extraction form consists of the following categories: characteristics of studies (eg, years, time of publication, and title), characteristics of participants, follow-up intervals, symptoms, and others. Regarding the extraction of symptom data at different follow-up times, we will follow these criteria: if a single study involves multiple follow-up points, we will sequentially extract the number of patients with post–COVID-19 condition and symptom data for each follow-up point that meets the inclusion criteria. Studies reporting follow-up times that do not align with the predetermined follow-up intervals (eg, 1 month or spanning multiple months or even a year) will be excluded. Additionally, studies must report the number of confirmed post–COVID-19 condition cases at each follow-up; those reporting only the baseline sample size will also be excluded. Discrepancies will be resolved through discussions until reaching consensus with another reviewer (BL).

Textbox 1. **Eligibility criteria for study inclusion in the systematic evaluation of symptom incidence in patients with post–COVID-19 condition.**

**Inclusion criteria**

- Document type: Original studies
- Study population: Adults (age≥18 years) after diagnosed with COVID-19
- Data availability: Providing specific details about the follow-up time (≥3 months), sample size, and symptom data of post–COVID-19 condition cases
- Study design: Observational study (cohort study, case-control study, cross-sectional study, and case series)
- Language: English publications

**Exclusion criteria**

- Document type: Reviews, basic experimental studies, and other unrelated papers
- Study population: Pediatric or adolescent populations
- Data availability: The study has a limited sample size (n≤30)
- Study design: Basic research, randomized controlled trials
- Language: Publications in other languages

### Risk of Bias Assessment

The same investigators (LC and NL) assessed the risk of bias for all qualifying studies based on the Joanna Briggs Institute critical appraisal tool for analytic study and cross-sectional study [23]. The investigators independently assessed and ranked the risk of bias for every eligible study as yes, no, unclear, and not applicable.

### Statistical Analysis

We conducted a meta-analysis (*metafor* package in RStudio, version 4.2.0; Posit PBC) using random effects models to estimate the prevalence of post–COVID-19 condition symptoms, aggregating data from multiple studies and incorporating study-specific effects into our analysis. The pooled effect results were presented along with their corresponding 95% CIs. To assess the proportion of variation attributable to between-study heterogeneity, analysis was conducted using *I*^2^ to quantify heterogeneity on a scale from 0% to 100%. In parallel, we performed a subgroup analysis based on the regional classification of the patients. Statistical significance for differences in prevalence among regions was determined using a threshold of *P*<.05. An extensive network analysis was performed to investigate the evolution of symptoms over 4 distinct time intervals, providing a comprehensive overview of the interrelations of symptom prevalence. We used Spearman intragroup methods to conduct correlation analysis, with the primary aim of identifying symptom pairs that exhibited statistically significant correlations (*P*<.05). In addition, fuzzy clustering was used to cluster symptoms across various follow-up intervals, providing insight into potential differences in clinical phenotypes among patients. The network was visualized using Cytoscape software (version 3.7.2; NRNB), providing a comprehensive and detailed illustration of symptom interactions over the designated time intervals.

## Results

### Overview

**Figure 1.**
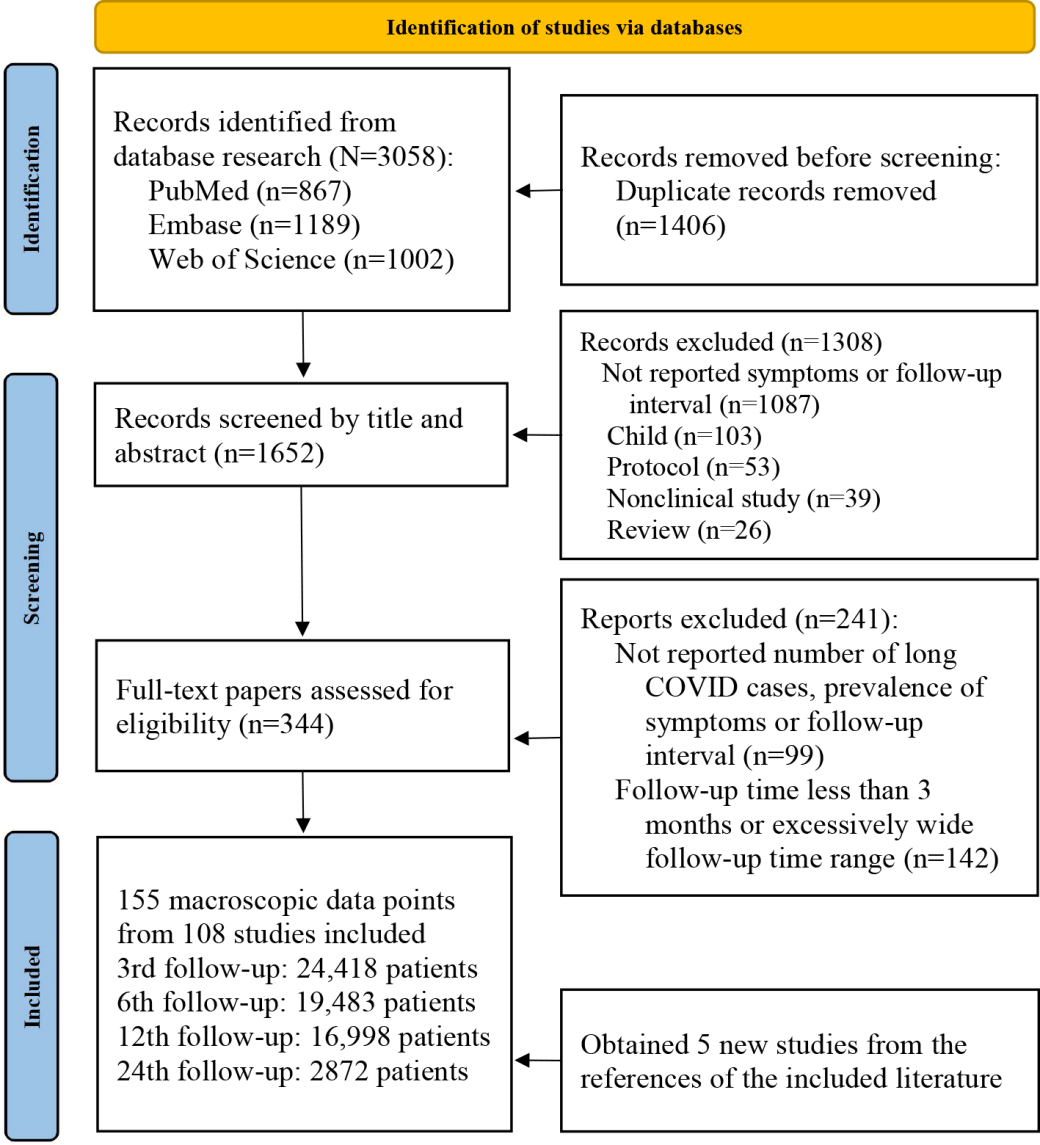
PRISMA (Preferred Reporting Items for Systematic Reviews and Meta-Analyses) flowchart of study selection for the systematic review on symptom prevalence of post–COVID-19 condition (December 2019-March 2025).

A systematic review of post–COVID-19 condition identified 3058 records from database searches. After removing duplicates, title or abstract screening yielded 344 potentially eligible papers. The full-text review excluded 241 papers (unmet inclusion criteria), with 5 additional studies identified through reference screening. A total of 108 studies were included in the qualitative synthesis, encompassing 63,771 confirmed cases. These studies reported 64 distinct symptoms spanning 9 organ systems, including respiratory, neurological, cardiovascular, and musculoskeletal manifestations. Neurological symptoms were the most frequent (22/64), followed by respiratory (11/64) and digestive symptoms (9/64). The flowchart of the review process is shown in Figure 1. The studies were mainly conducted in Europe (55/108), Asia (38/108), America (13/108), and Africa (2/108). The number of patients and symptoms decreased over time, with neurological symptoms being the most prevalent. The characteristics of the studies are summarized in Table 1. The symptom classification system after standardization using *ICD-11* is presented in Table 2. The results of the quality assessment of the included studies are presented in Table S2 in Multimedia Appendix 1 [24-131].

**Table 1.** Summary of basic characteristics of included studies on post–COVID-19 condition, including study design, geographic location, symptom numbers, and follow-up period.

		All studies		3rd month		6th month		12th month		24th month	
		Studies, n	Patients, n	Studies, n	Patients, n	Studies, n	Patients, n	Studies, n	Patients, n	Studies, n	Patients, n
Overall^a^		108	63,771	43	24,418	51	19,483	50	16,998	11	2872
Study type											
	Prospective cohort study	35	17,894	15	11,505	17	2766	19	3027	5	596
	Cross-sectional study	23	20,364	10	7689	9	6259	6	5993	1	423
	Cohort study	30	18,122	9	4305	17	5795	17	6379	4	1643
	Prospective observational study	8	4835	3	194	4	3794	2	637	1	210
	Retrospective cohort study	7	1276	4	390	2	682	3	204	—^b^	—
	Retrospective observational study	4	1145	2	335	2	187	2	623	—	—
	Case control	1	135	—	—	—	—	1	135	—	—
Regions											
	Europe	55	34,433	19	12,886	24	8358	28	11,831	5	1358
	Asia	38	24,727	16	9023	18	9746	14	4484	5	1474
	America	13	3149	6	1189	8	1308	7	612	1	40
	Africa	2	1462	2	1320	1	71	1	71	—	—

a Since multiple follow-up dates were reported in 31 clinical studies, the total number of follow-up periods exceeds 99.

b Not available.

**Table 2.** Standardize and normalize symptoms using the *International Classification of Diseases, 11th Revision* (*ICD-11*) and classify all reported symptoms in the study into different systems.

Symptom-based disease system	Symptoms, n				
	All studies	3rd month	6th month	12th month	24th month
Nervous	23	22	18	19	16
Respiratory	11	10	9	8	9
Digestive	9	9	6	6	5
Circulatory	2	2	2	2	3
Musculoskeletal	5	4	3	4	3
Endocrine	3	3	2	1	—^a^
Skin	5	3	4	4	5
Urogenital	3	2	1	1	—
Other	3	3	3	3	2
Overall	64	60	50	50	45

a Not available.

### Prevalence of Clinical Symptoms

Meta-analysis of symptom incidence rates at different follow-up time points revealed distinct dynamic evolution characteristics. At the third-month follow-up, 43 studies reported on 60 clinical symptoms in a total of 24,418 patients. Fatigue (52%, 95% CI 45%‐59%) and dyspnea (36%, 95% CI 29%‐43%) were the most common symptoms, while neurological and psychiatric symptoms such as weakness in the limbs (36%, 95% CI 20%‐53%) and anxiety (31%, 95% CI 17%‐44%) had already begun to emerge. Additionally, there are 56 other symptoms (attention deficit, myalgia, insomnia, hair loss, and cough) with an incidence rate of less than 30% (Figure S1A in Multimedia Appendix 1). A total of 51 studies (n=19,483) reported the prevalence rates of 50 symptoms at the sixth-month follow-up. Although fatigue (48%, 95% CI 42%‐54%) remained the most prevalent symptom, dyspnea (31%, 95% CI 26%‐36%) showed a declining trend, and neurological and psychiatric symptoms (anxiety: 24% and insomnia: 23%) persisted. At the 12th-month follow-up, 50 studies with a combined total of 16,998 patients detailed the prevalence rates of 50 symptoms. Beyond the persistently high prevalence of fatigue (46%, 95% CI 41%‐52%), dyspnea has declined to the fourth most reported symptom, while neurological manifestations (attention deficit: 32%, 95% CI 22%‐43%; memory impairment: 28%, 95% CI 19%‐37%; and insomnia: 28%, 95% CI 21%‐35%) and cardiovascular symptoms (arrhythmia: 29%, 95% CI 12%‐46%) have emerged as more prominent clinical features. At the 2-year follow-up, the prevalence rates of 45 symptoms were reported across 11 studies involving 2872 patients. Fatigue remained the most prevalent symptom (54%, 95% CI 45%‐64%), with neurological manifestations (particularly memory deficit at 42%, 95% CI 28%‐56%) constituting the predominant clinical presentation. Notably, emerging symptoms including gastrointestinal bloating (45%, 95% CI 38%‐51%) and respiratory nasal congestion (38%, 95% CI 32%‐45%) have become increasingly apparent. Please refer to Figure S1 in Multimedia Appendix 1 for a detailed illustration of forest plots of symptom prevalence at different follow-ups.

**Figure 2.**
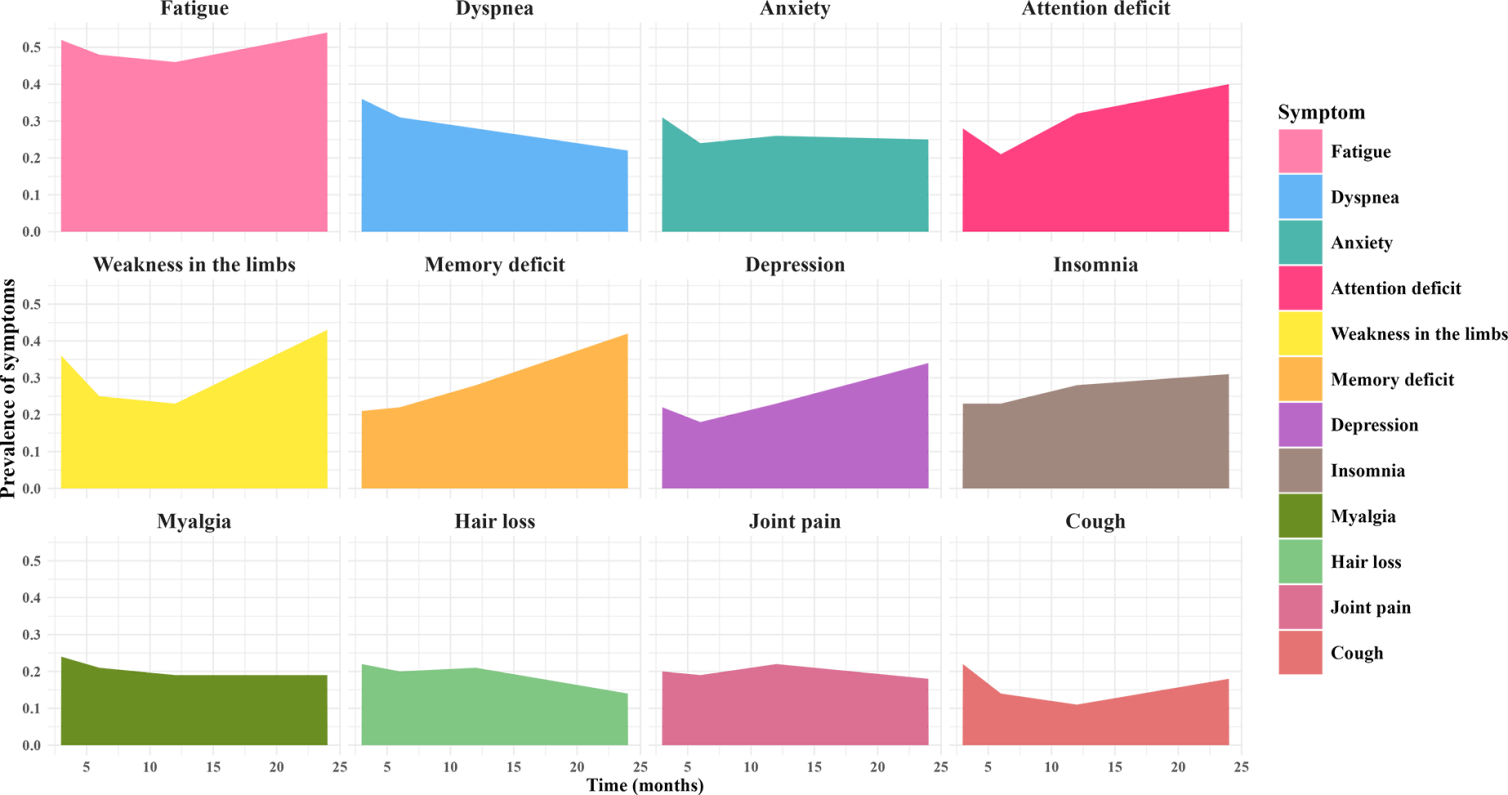
Trends in the prevalence of major post–COVID-19 condition symptoms across 4 follow-up time points (3rd, 6th, 12th, and 24th month) in studies conducted between December 2019 and March 2025, highlighting the progression of symptoms with fatigue as the core symptom and the shift from respiratory involvement to multisystem dysfunction.

Overall, fatigue consistently ranked as the most prevalent symptom, particularly prominent among patients with post–COVID-19 condition. Moreover, neurological symptoms demonstrated a progressive worsening trend. Most importantly, as the disease duration extended, the symptom spectrum shifted from a predominance of respiratory symptoms in the early stages to multisystem involvement, including cardiovascular, digestive, and neurological systems, ultimately forming a complex clinical presentation characterized by fatigue as the core feature and multisystem dysfunction at the 2-year follow-up. The prevalence rates of the 12 most frequently reported symptoms across the 4 follow-up time points are presented in Figure 2.

### Subgroup Analysis

To account for the comprehensive impact of temporal and spatial dynamics on symptom prevalence, we conducted a subgroup analysis based on the geographical locations of the studies. The results revealed notable differences in symptom prevalence across different regions. Overall, Africa reported the fewest symptoms, while Asia and Europe reported the highest number of symptoms. The Americas focused more on symptom incidence within 1 year. At the third-month follow-up, fatigue (68%, 95% CI 50%‐85%) and dyspnea (56%, 95% CI 15%‐98%) had the highest prevalence rates in Africa. However, insufficient attention to other symptoms has resulted in limited data availability, making further symptom comparisons challenging. The Americas exhibited a distinct neuromusculoskeletal symptom profile during short-term follow-up, with significantly higher prevalence rates of anxiety (60%, 95% CI 54%‐66%) and depression (36%, 95% CI 16%‐55%) compared to other regions. Notably, joint pain (51%, 95% CI 33%‐68%) and weakness in the limbs (57%) were particularly prominent. Over time, both anxiety and joint pain maintained persistently high prevalence rates, reflecting a substantial long-term disease burden. During early follow-up periods, Asian cohorts demonstrated the highest reported symptom diversity (n=58 distinct manifestations) while exhibiting comparatively lower prevalence rates of dyspnea relative to other continental populations. This epidemiological pattern suggests a milder degree of respiratory involvement in Asian patient populations during the acute-postacute transition phase. Limb weakness was more commonly observed initially, but gradually diminished over time, evolving into a predominantly neurological symptom profile characterized by insomnia, depression, and memory impairment. The European region exhibited a more complex symptom spectrum during long-term follow-up, including an increasing prevalence of fatigue (66%, 95% CI 52%‐79%) and worsening cognitive dysfunction. Additionally, a range of new-onset symptoms emerged, such as bloating (45%) and other multisystem manifestations. Concrete details are provided in Table S3 in Multimedia Appendix 1.

### Association and Clustering Analysis of Symptoms

We conducted the Spearman rank correlation analysis of symptoms across different follow-up periods, identifying positively correlated symptom pairs with statistically significant differences. These correlations were visualized using Cytoscape to display the core symptom clusters for each follow-up period.

**Figure 3.**
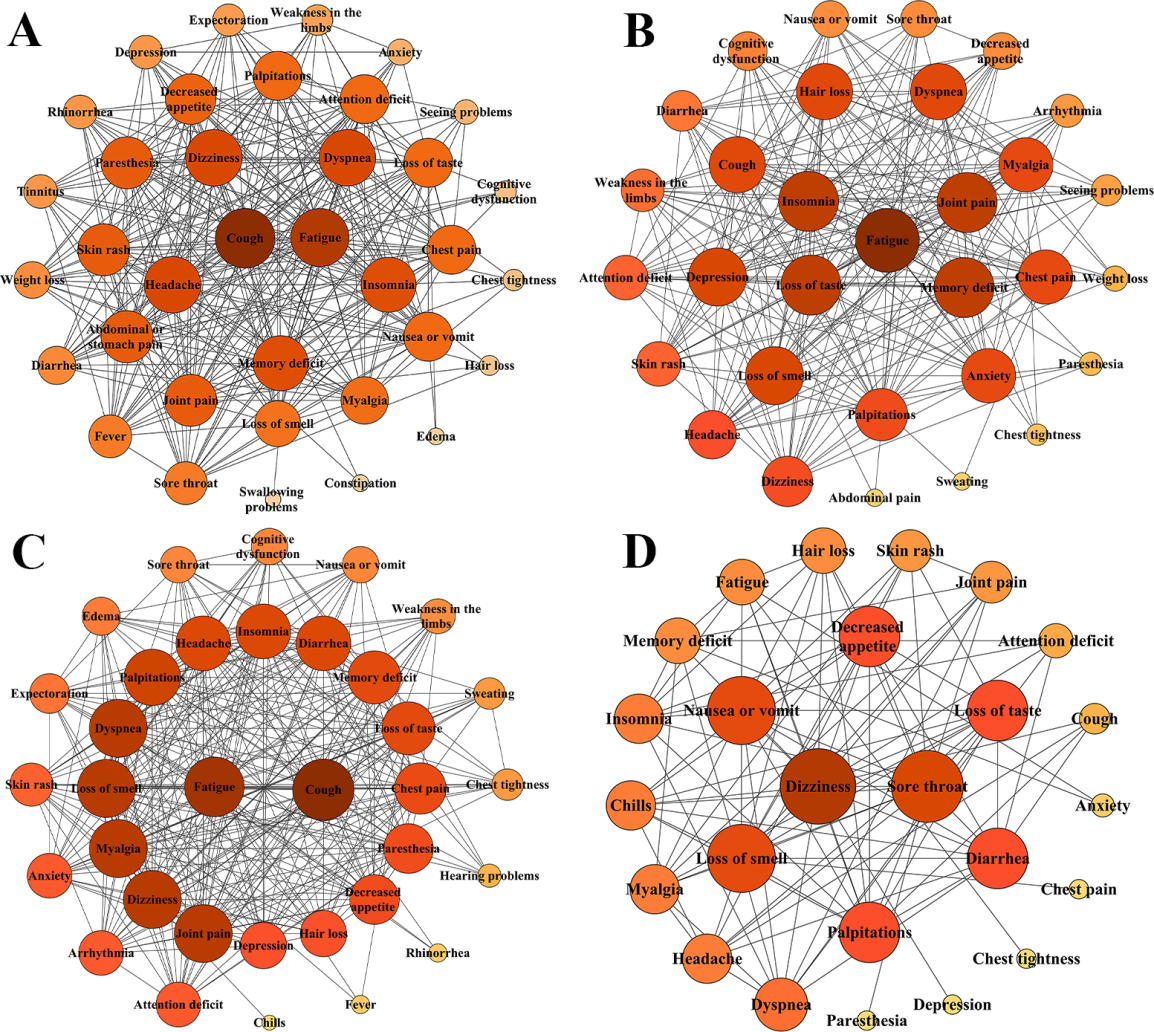
Symptom network diagrams of post–COVID-19 condition at different follow-up time points showing symptom interactions and core nodes. Longitudinal symptom network analysis revealed a transition from respiratory-neurological symptom clusters to chronic, multisystem symptoms over time. (A) 3rd-month follow-up, (B) 6th-month follow-up, (C) 12th-month follow-up, and (D) 24th-month follow-up; nodes represent individual symptoms and edges to illustrate the correlations between symptoms for each interval. Each node’s color and size represent the degree of the symptom, with darker colors and larger size indicating a higher number of symptoms correlated with it.

The third-month follow-up analysis revealed significant positive correlations between core symptoms (cough, fatigue, headache, dizziness, and dyspnea) and other persistent symptoms. Network analysis demonstrated a complex symptom interaction pattern during early follow-up, comprising 36 distinct symptoms (Figure 3A). Cough showed the highest closeness centrality (0.875), node degree (Degree=30), and significantly elevated stress centrality (424) compared to other symptoms, indicating its critical role in symptom propagation pathways. Notably, fatigue, dyspnea, dizziness, and headache demonstrated comparable closeness centrality and topological coefficients, suggesting early stage respiratory-neurological system interactions. The network analysis at the sixth-month follow-up identified a core symptom cluster consisting of fatigue, memory impairment, ageusia, arthralgia, and insomnia. Fatigue demonstrated significantly higher betweenness centrality and closeness centrality compared to other symptoms, with the highest node strength in the entire network (Degree=26), indicating its pivotal role as a central hub connecting multiple symptom subgroups. Comparative analysis revealed a progressive reduction in symptom complexity, with the number of nodes decreasing from 36 at 3 months to 31 at 6 months. Notably, respiratory-related acute symptoms (eg, dyspnea and chest pain) became marginalized in the network topology. The neurocognitive-somatic symptom cluster, including memory impairment, arthralgia, insomnia, and ageusia, showed converging topological coefficients (0.614-0.625), suggesting that these symptoms may share neural network connectivity, potentially reflecting common neuroinflammatory mechanisms. The 12th-month follow-up network analysis revealed a newly formed core symptom cluster consisting of fatigue, cough, dizziness, anosmia, myalgia, and arthralgia. Dizziness emerged as the most central symptom, exhibiting the highest betweenness centrality (0.067) and stress centrality (322) in the network, along with a closeness centrality of 0.846. Fatigue maintained its prominent position with equally high betweenness centrality (0.067) and significant stress centrality (322), confirming both symptoms as critical information hubs and key propagation nodes within the symptom network. Notably, myalgia and arthralgia demonstrated nearly identical topological coefficients (0.645), suggesting shared immunoinflammatory pathway involvement. The analysis further identified cough as having the highest closeness centrality (0.891) in the entire network while showing topological coefficient similarity with anosmia (0.61), potentially indicating common inflammatory pathway mechanisms underlying these symptom pairs. Comparative analysis revealed a significant reduction in network complexity at a 2-year follow-up, with the number of nodes decreasing to 25 and the complete disappearance of respiratory symptoms (eg dyspnea and chest pain) and acute inflammatory manifestations (fever). Dizziness maintained its network dominance, exhibiting persistently high betweenness centrality (0.189) and closeness centrality (0.75) while achieving the highest node degree (Degree=16), indicating substantial strengthening of its role as the primary information hub. In contrast, cough exited the core cluster, demonstrating marked reductions in both node degree (to 4) and stress centrality (to 14), reflecting its substantially diminished role in symptom propagation. Gastrointestinal and cardiovascular manifestations emerged as increasingly prominent over time, demonstrating that post–COVID-19 condition follows a characteristic evolutionary trajectory culminating in chronic, persistent multisystem involvement. Complete symptom network analysis results appear in Table S4 in Multimedia Appendix 1.

Furthermore, we conducted a clustering analysis on longitudinal symptom prevalence data, which revealed 2 distinct clinical subtypes of post–COVID-19 condition. To avoid subjective severity-based labeling, we adopted descriptive terms based on symptom distribution patterns and classified the clusters as the “dispersed type” and the “concentrated type.” The dispersed type (cluster 1) was characterized by a broader diversity of symptoms with generally lower prevalence rates across multiple organ systems. In contrast, the concentrated type (cluster 2) showed a narrower symptom spectrum but with higher prevalence rates, predominantly involving neurological and respiratory systems. This clustering analysis finding was most pronounced within 1 year of follow-up. At the third-month follow-up, notable differences in symptom distribution were evident (Multimedia Appendix 3): cluster 1 showed a wide (n=59) but lower-range distribution of symptom prevalence (ranging from 0.5% to 20%), whereas cluster 2 demonstrated a narrower (n=39) but higher-range distribution (from 20% to 50%), indicating a more focused and severe symptom profile. The different colors in the legend represent different systems, with symptoms of the same system shown in the same area. The letter “n” represents the number of macro data points, and the percentages indicate the average prevalence of symptoms within each cluster. These findings suggest 2 distinct trajectories of post–COVID-19 condition: one subtype characterized by chronic, multisystem, and persistent symptoms (cluster 1), and another with concentrated, inflammation-related symptoms of greater severity (cluster 2). The longitudinal analysis further revealed a progressive convergence in the number of reported symptoms between the 2 clusters over time, suggesting a potential evolutionary trend toward more chronic and diffuse symptomatology in the postacute phase of SARS-CoV-2 infection. Figure S2 in Multimedia Appendix 1 illustrates the 6th- and 12th-month follow-up findings.

## Discussion

### The Complex Network Dynamics of Long COVID Symptoms

The intricate interactions of post–COVID-19 condition symptoms are reflected in their dynamically evolving network characteristics. During the early follow-up phase (third month), respiratory symptoms (cough and dyspnea) showed significant correlations with neurological manifestations (headache and dizziness), suggesting potential shared pathways linking respiratory inflammation and neurological dysfunction, likely attributable to persistent multisystem impacts of residual viral components from the acute infection phase. Current evidence highlights autonomic dysfunction, particularly manifesting as postural orthostatic tachycardia syndrome–like symptoms (postural orthostatic tachycardia, dizziness, and gastrointestinal dysregulation), as a defining pathophysiological characteristic of post–COVID-19 condition. The proposed mechanisms include direct viral neurotropism, immune-mediated autonomic nerve injury, and chronic inflammatory modulation of autonomic regulatory circuits [132-134]. As the disease progressed, fatigue emerged as a persistent central node connecting multiple symptom clusters, with its centrality becoming particularly prominent at 6‐12 months, demonstrating strong associations with neurocognitive symptoms (memory impairment and insomnia) and musculoskeletal symptoms (arthralgia and myalgia). This pattern may reflect chronic neuroinflammation or autoantibody-mediated damage mechanisms. Previous studies have similarly identified that post–COVID-19 condition is characterized by persistent immune dysregulation, manifesting as chronic inflammation and sustained immune activation following acute infection [135]. The underlying pathogenic mechanisms may include viral antigen persistence, autoimmune responses, and cytokine-mediated tissue damage, which collectively contribute to its multisystem clinical manifestations. Autoimmunity has been specifically implicated as a driver of symptoms such as joint pain and neurological manifestations, while prolonged immune activation may lead to progressive tissue injury and chronic symptomatology [136-139]. Of particular note, by the 2-year follow-up, the symptom network complexity had significantly diminished, with respiratory manifestations having largely resolved, while neurological, circulatory, and digestive system symptoms (including dizziness, nausea, vomiting, and palpitations) emerged as the predominant clinical features. Some researchers have proposed the hypothesis that the gut-brain axis constitutes a bidirectional neuro-immune-endocrine network, with vagal-mediated signaling and microbial metabolite production (particularly serotonin, predominantly gut-derived) critically modulating both enteric and central nervous system functions. Mounting evidence indicates that gut dysbiosis and impaired microbiome-immune crosstalk contribute to neuropsychiatric and systemic manifestations [140,141]. Another key hypothesis posits that vascular compromise leads to tissue hypoperfusion and hypoxia, particularly affecting high-metabolic organs (brain, heart, and skeletal muscle), potentially explaining fatigue, cognitive impairment, and myalgia [142-144]. Thus, it can be seen that the pathophysiology of long COVID is characterized by a multifactorial interplay of immune dysregulation, persistent vascular or organ injury, chronic inflammation, and neuroendocrine dysfunction, resulting in heterogeneous clinical manifestations [145-147]. This complex pathophysiology complicates mechanistic elucidation while accounting for the condition’s diverse clinical phenotypes.

### Regional Variations in Symptom Reporting and Underlying Factors

The study identified significant regional variations in symptom reporting, likely driven by multiple interacting factors. Disparities in health care resource distribution play a critical role—well-established follow-up systems in Europe and North America may capture a broader spectrum of symptoms, whereas Africa’s constrained health care infrastructure and overburdened medical service systems may contribute to both significant underreporting of symptoms and elevated prevalence of primary clinical manifestations [148]. Genetic background differences also contribute; for instance, the high prevalence of arthralgia in the Americas may be linked to specific human leukocyte antigen genotypes [149,150]. Divergent vaccination strategies, particularly the varying protective effects of mRNA versus inactivated vaccines, might modulate immune responses and influence long-term symptom profiles. The spatiotemporal distribution of viral variants is another key factor, as the study period spanned both the Delta and Omicron waves. The Delta variant may induce long-term sequelae characterized by hyperinflammatory or neurotropic pathological patterns [151], whereas Omicron infection appears to confer a lower risk of developing post–COVID-19 condition [152]. Additionally, cultural factors, such as differing perceptions of mental health, may lead to underreporting of psychiatric symptoms (eg, anxiety) in Asian populations. These variations underscore the need for a globally standardized core symptom assessment tool and careful consideration of contextual factors when interpreting cross-regional findings.

### Symptom Dynamics and Targeted Management

Fatigue, as a common nonspecific symptom, frequently overlaps with various other clinical manifestations, complicating the identification of condition-specific patterns. Notably, network analysis based on symptom prevalence suggests that cough and dyspnea may serve as key sentinel symptoms for early detection of post–COVID-19 condition. Within the context of long-term sequelae, symptoms such as dizziness, nausea or vomiting, and palpitations play a critical role in its recognition and diagnosis. The nonspecific nature of fatigue and its frequent overlap with diverse clinical manifestations complicate the identification of disease-specific patterns. Symptom prevalence–based network analysis highlights cough and dyspnea as potential sentinel symptoms for early recognition of post–COVID-19 condition, while neuro-circulatory-gastroenteric symptom clusters (particularly dizziness, nausea or vomiting, and palpitations) emerge as critical diagnostic markers in the chronic phase. This evolving understanding warrants dynamic, phase-adapted management approaches: early-stage interventions should prioritize respiratory rehabilitation and inflammatory response modulation, whereas chronic-phase care requires focused neurological symptom management integrated with multidisciplinary strategies addressing concomitant digestive and circulatory system involvement. Regionally tailored public health strategies are essential, exemplified by musculoskeletal symptom–focused interventions in the Americas and enhanced cognitive rehabilitation frameworks in Europe, reflecting geographical variations in disease manifestation and health care system capacities.

### Study Limitations and Future Directions

With regard to sampling constraints, the limited representation of data from Africa and certain parts of Southeast Asia may restrict the global generalizability of our conclusions, as region-specific symptom patterns or subtypes may remain unidentified. This regional imbalance could also introduce bias, as symptom prevalence and reporting practices are likely influenced by local health care infrastructure, follow-up systems, and cultural perceptions of illness. Moreover, the included studies largely lacked adjustment for socioeconomic determinants of health. Given that many countries have lifted containment measures, resumed economic activities, and reopened borders, this global shift toward adaptive coexistence with SARS-CoV-2 may lead to systematic underreporting of symptoms, especially mild or chronic manifestations. Additionally, the predominant reliance on passive follow-up methods may have missed clinically relevant but less severe cases, further contributing to data skewness. Another notable limitation is the absence of variant-specific data. The study period spans multiple waves of the pandemic, including those driven by the Delta and Omicron variants, which have been associated with distinct clinical trajectories. However, due to data availability constraints, we were unable to stratify symptom trajectories by viral strain, limiting the capacity to attribute observed patterns to specific viral characteristics. These limitations underscore the need for future studies to adopt a more standardized, comprehensive, and globally inclusive approach. We recommend prospective cohort studies with individual-level, longitudinal follow-up, stratified by region, socioeconomic status, and viral variant. Active surveillance in underrepresented regions, combined with systematic collection of social determinants and clinical metadata, is essential to generate more robust, equitable, and actionable insights into post–COVID-19 condition.

### Conclusions

Building upon these findings, a comprehensive synthesis of post–COVID-19 condition research reveals a complex and evolving clinical landscape. Across varying follow-up periods encompassing diverse symptoms and affected organ systems, we demonstrate that the chronic and persistent effects of post–COVID-19 condition pose significant challenges for both patients and health care providers. Symptom prevalence exhibits temporal fluctuations, with neurological manifestations emerging as the disease’s most prominent feature. In regions with limited research coverage, such as Africa, greater attention should be directed toward understanding specific symptom patterns and disease progression. Consequently, the establishment of a stratified management system based on disease staging and regional characteristics becomes imperative to address this heterogeneous condition. However, given the limitations of population-level data, our findings should be used as a guideline for understanding the broader trends of post–COVID-19 condition, but clinical decisions should be personalized, taking into account each patient’s unique characteristics. Future studies incorporating individual-level data are needed to provide more granular insights and inform precision medicine strategies for post–COVID-19 care.

## Supplementary material

10.2196/72221 Multimedia Appendix 1 Detailed data and analyses on symptom characteristics and analytical findings in post–COVID-19 condition.

10.2196/72221 Multimedia Appendix 2 Search strategy.

10.2196/72221 Multimedia Appendix 3 Clustering analysis of post–COVID-19 condition symptoms by prevalence at the third-month follow-up.

10.2196/72221 Checklist 1 PRISMA (Preferred Reporting Items for Systematic Reviews and Meta-Analyses) checklist.

## Abbreviations

*ICD-11*: *International Classification of Diseases, 11th Revision*
MeSH: Medical Subject Headings
PRISMA: Preferred Reporting Items for Systematic Reviews and Meta-Analyses
