# Supplementary material for: Symptom Trajectories and Clinical Subtypes in Post–COVID-19 Condition: Systematic Review and Clustering Analysis
Source: JMIR Public Health Surveill. 2025 Jul 18;11:e72221. doi: 10.2196/72221 (PMC12296217; doi:10.2196/72221)
Supplement: Multimedia Appendix 1 [file publichealth-v11-e72221-s001.doc]

**Table S1 Complete list of symptoms and their corresponding system classifications of post COVID-19 condition**

| **Systems** | **Symptoms** |
| --- | --- |
| **Respiratory** | chest pain, chest tightness, dyspnea, expectoration, cough, nasal congestion, rhinorrhea, sneezing, sore throat, itchy throat, hoarse voive |
| **Nervous** | abnormal walking posture, attention deficit, memory deficit, anxiety, cognitive dysfunction, fainting, hallucinations, headache, insomnia, paraesthesia, seizures, loss of smell, loss of taste, tinnitus, tremor, depression, dizziness, fear of heat, hearing problems, seeing problems, earache, chills, fatigue |
| **Circulatory** | arrhythmia, palpitations |
| **Digestive** | abdominal pain, bitterness in the mouth, bloating, constipation, nausea/vomit, swallowing problems, thirst, decreased appetite, diarrhea |
| **Musculoskeletal** | joint pain, myalgia, twitching of extremities, arthritis, weakness in the limbs |
| **Endocrine** | dysmenorrhea, menstrual irregularities, weight loss |
| **Skin** | discoloration of toes and fingers, skin lesions, hair loss, skin rash, sweating |
| **Urogenital** | sexual dysfunction, urination problems, edema |
| **Other** | fever, red eye, sore eyes |

**Table S2 Risk of bias assessment of included studies**

**Table S2A** JBI Critical Appraisal Checklist for cohort studies

|  | Were the two groups similar and recruited from the same population? | Were the exposures measured similarly to assign people to both exposed and unexposed groups? | Was the exposure measured in a valid and reliable way? | Were confounding factors identified? | Were strategies to deal with confounding factors stated? | Were the groups/participants free of the outcome at the start of the study (or at the moment of exposure)? | Were the outcomes measured in a valid and reliable way? | Was the follow up time reported and sufficient to be long enough for outcomes to occur? | Was follow up complete, and if not, were the reasons to loss to follow up described and explored? | Were strategies to address incomplete follow up utilized? | Was appropriate statistical analysis used? |
| --- | --- | --- | --- | --- | --- | --- | --- | --- | --- | --- | --- |
| Kim et al (2024)^24^ | Yes | Yes | Yes | No | No | Yes | Yes | Yes | Unclear | No | No |
| Mora et al (2022)^25^ | Yes | Yes | Yes | Yes | No | Yes | No | Yes | Unclear | No | Yes |
| Salvador et al (2022)^26^ | Yes | Yes | Yes | Yes | Yes | Yes | Yes | Yes | Unclear | No | Yes |
| Catalán et al (2021)^27^ | Yes | Yes | Yes | No | No | Not applicable | No | Yes | Unclear | No | No |
| Messin et al (2021)^28^ | Yes | Yes | Yes | Yes | No | Not applicable | Yes | Yes | Unclear | Yes | Yes |
| Babicki et al (2023)^29^ | Yes | Yes | Yes | Yes | Yes | Not applicable | Not applicable | Yes | Unclear | No | Yes |
| Sykes et al (2023)^30^ | Yes | Yes | Yes | Yes | Yes | Not applicable | Yes | Yes | Unclear | No | Yes |
| Serrano et al (2023)^31^ | Yes | Yes | Yes | Yes | Yes | Not applicable | No | Yes | Unclear | Yes | Yes |
| Huang et al (2022)^32^ | Yes | Yes | Yes | Yes | Yes | Not applicable | Yes | Yes | Unclear | Yes | Yes |
| Villa et al (2024)^33^ | Yes | Yes | Yes | Yes | Yes | Not applicable | No | Yes | Unclear | No | Yes |
| Amorim et al (2022)^34^ | Yes | Yes | Yes | Yes | Yes | Yes | No | Yes | Unclear | No | Yes |
| Becker et al (2021)^35^ | Yes | Yes | No | Yes | Yes | Not applicable | Yes | Yes | Unclear | Yes | Yes |
| Rigoni et al (2022)^36^ | Yes | Yes | Yes | Yes | Yes | Yes | Yes | Yes | Unclear | No | Yes |
| Blomberg et al (2021)^37^ | Yes | Yes | Yes | Yes | Yes | Not applicable | Yes | Yes | Unclear | No | Yes |
| González et al (2022)^38^ | Yes | Yes | Yes | Yes | Yes | Yes | No | Yes | Unclear | Yes | Yes |
| Yaksi et al (2022)^39^ | Yes | Yes | Yes | Yes | Yes | Not applicable | No | Yes | Unclear | Yes | Yes |
| Adler et al (2022)^40^ | Yes | Yes | Yes | Yes | Yes | Not applicable | No | Yes | Unclear | No | Yes |
| Lombardo et al (2021)^41^ | Yes | Yes | Yes | Yes | Yes | Yes | No | Yes | Unclear | No | Yes |
| Kim et al (2022)^42^ | Yes | Yes | Yes | No | No | Yes | No | Yes | Unclear | No | No |
| Bhandari et al (2023)^43^ | Yes | Yes | No | Yes | No | Yes | Yes | Yes | Yes | Yes | No |
| Fang et al (2021)^44^ | Yes | Yes | Yes | Yes | Yes | Not applicable | No | Yes | Unclear | Yes | Yes |
| Maaden et al (2022)^45^ | Yes | Yes | Yes | Yes | Yes | Not applicable | Yes | Yes | Unclear | No | Yes |
| Hossain et al (2021)^46^ | Yes | Yes | Yes | Yes | Yes | Yes | Yes | Yes | Unclear | Yes | Yes |
| Canales et al (2022)^47^ | Yes | Yes | Yes | Yes | Yes | Yes | Yes | Yes | Yes |  | Yes |
| Zhang et al (2023)^48^ | Yes | Yes | No | Yes | Yes | Yes | Yes | Yes | Unclear | No | Yes |
| Shrestha et al (2024)^49^ | Yes | Yes | Yes | Yes | No | Yes | No | Yes | Unclear | No | No |
| Perisse et al (2023)^50^ | Yes | Yes | Yes | Yes | Yes | Yes | Yes | Yes | Unclear | No | Yes |
| Huang et al (2020)^51^ | Yes | Yes | No | Yes | Yes | Not applicable | Yes | Yes | Unclear | Yes | Yes |
| Maestrini et al (2021)^52^ | Yes | Yes | Yes | Yes | Yes | Yes | Yes | Yes | Yes |  | Yes |
| Li et al (2022)^53^ | Yes | Yes | Yes | Yes | Yes | Yes | Yes | Yes | Unclear | Yes | Yes |
| Zhao et al (2021)^54^ | Yes | Yes | No | Yes | Yes | Yes | Yes | Yes | Unclear | No | Yes |
| Peghin et al (2023)^55^ | Yes | Yes | Yes | Yes | Yes | Yes | No | Yes | Unclear | No | Yes |
| Kirchberger et al (2022)^56^ | Yes | Yes | No | No | No | Not applicable | No | Yes | Unclear | No | No |
| David et al (2022)^57^ | Yes | Yes | Yes | No | No | Not applicable | No | Yes | Unclear | Yes | No |
| Zhang et al (2021)^58^ | Yes | Yes | No | Yes | Yes | Not applicable | No | Yes | Unclear | Yes | Yes |
| Millet et al (2022)^59^ | Yes | Yes | No | No | No | Yes | No | Yes | Unclear | No | No |
| Liu et al (2022)^60^ | Yes | Yes | Yes | Yes | No | Yes | Yes | Yes | Unclear | Yes | No |
| Wahlgren et al (2023)^61^ | Yes | Yes | Yes | Yes | No | Yes | Yes | Yes | Unclear | No | No |
| Arnold et al (2020)^62^ | Yes | Yes | Yes | Yes | No | Yes | Yes | Yes | Unclear | No | No |
| Berentschot et al (2024)^63^ | Yes | Yes | Yes | Yes | No | Yes | Yes | Yes | Unclear | Yes | No |
| Rocha et al (2024)^64^ | Yes | Yes | Yes | Yes | No | Yes | Yes | Yes | Unclear | No | No |
| Zhang et al (2025)^65^ | Yes | Yes | Yes | Yes | No | Yes | Yes | Yes | Unclear | No | No |

**Table S2B** JBI Critical Appraisal Checklist for Case Series

|  | Were there clear criteria for inclusion in the case series? | Was the condition measured in a standard, reliable way for all participants included in the case series? | Were valid methods used for identification of the condition for all participants included in the case series? | Did the case series have consecutive inclusion of participants? | Did the case series have complete inclusion of participants? | Was there clear reporting of the demographics of the participants in the study? | Was there clear reporting of clinical information of the participants? | Were the outcomes or follow up results of cases clearly reported? | Was there clear reporting of the presenting site(s)/clinic(s) demographic information? | Was statistical analysis appropriate? |
| --- | --- | --- | --- | --- | --- | --- | --- | --- | --- | --- |
| Martino et al (2022)^66^ | Yes | Yes | Yes | Yes | Yes | No | Yes | Yes | Yes | Yes |
| Huang et al (2021)^67^ | Yes | Yes | No | Unclear | Unclear | Yes | Yes | Yes | Yes | Yes |
| Eldin et al (2024)^68^ | Yes | Yes | Yes | No | No | No | No | Yes | Yes | Yes |
| Nayani et al (2023)^69^ | No | Yes | No | No | No | Yes | No | Yes | Yes | Yes |
| PHOSP-COVID et al (2022)^70^ | Yes | Yes | No | Unclear | Unclear | Yes | No | Yes | No | Yes |
| Straudi et al (2022)^71^ | Yes | Yes | Yes | Unclear | Unclear | Yes | Yes | Yes | Yes | No |
| Malesevic et al (2023)^72^ | No | Yes | Yes | Unclear | Unclear | Yes | Yes | Yes | Yes | Yes |
| Karaarslan et al (2022)^73^ | Yes | Yes | No | Unclear | Unclear | Yes | Yes | Yes | No | Yes |
| Seang et al (2022)^74^ | Yes | Yes | Yes | Unclear | Unclear | No | Yes | Yes | Yes | Yes |
| Martinez et al (2021)^75^ | Yes | Yes | No | Unclear | Unclear | No | Yes | Yes | Yes | Yes |
| Silva et al (2023)^76^ | Yes | Yes | No | Unclear | Unclear | No | Yes | Yes | Yes | Yes |
| Comelli et al (2022)^77^ | Yes | Yes | Yes | Yes | Yes | Yes | Yes | Yes | Yes | Yes |
| Och et al (2021)^78^ | Yes | Yes | Yes | Unclear | Unclear | Yes | Yes | Yes | Yes | Yes |
| Seeßle et al (2021)^79^ | Yes | Yes | Yes | Unclear | Unclear | No | Yes | Yes | Yes | Yes |
| Tok et al (2024)^80^ | Yes | Yes | No | Unclear | Unclear | Yes | Yes | Yes | Yes | Yes |
| Smith et al (2023)^81^ | Yes | Yes | No | Unclear | No | Yes | No | Yes | Yes | Yes |
| Monteiro et al (2023)^82^ | Yes | Yes | Yes | Unclear | Unclear | Yes | No | Yes | No | Yes |
| Shah et al (2022)^83^ | Yes | No | No | Unclear | Unclear | Yes | No | Yes | Yes | Yes |
| Augustin et al (2021)^84^ | No | Yes | No | Unclear | Unclear | Yes | Yes | Yes | Yes | Yes |
| Dryden et al (2022)^85^ | Yes | Yes | Yes | Unclear | Unclear | Yes | Yes | Yes | Yes | Yes |
| Malinowska et al (2021)^86^ | Yes | Yes | Yes | Unclear | Unclear | Yes | Yes | Yes | No | Yes |
| Hyassat et al (2023)^87^ | No | Yes | Yes | No | No | Yes | Yes | Yes | Yes | No |
| Xiong et al (2021)^88^ | Yes | Yes | No | Unclear | Unclear | Yes | Yes | Yes | Yes | No |
| Onieva et al (2024)^89^ | Yes | No | No | Unclear | Unclear | Yes | Yes | Yes | Yes | No |
| Gsapar et al (2023)^90^ | Yes | Yes | Yes | Unclear | Unclear | Yes | Yes | Yes | Yes | Yes |
| Guliani et al (2023)^91^ | Yes | Yes | Yes | No | No | No | Yes | Yes | Yes | No |
| Lapa et al (2023)^92^ | Yes | Yes | No | Unclear | Unclear | Yes | Yes | Yes | Yes | Yes |
| Kalak et al (2022)^93^ | No | Yes | No | No | No | No | Yes | Yes | Yes | Yes |
| Muz et al (2023)^94^ | No | Yes | No | Unclear | Unclear | Yes | No | Yes | Yes | No |
| Antoni et al (2023)^95^ | No | Yes | Yes | No | No | Yes | Yes | Yes | Yes | Yes |
| Emecen et al (2023)^96^ | No | Yes | No | Unclear | Unclear | Yes | Yes | Yes | Yes | Yes |
| Méndez et al (2021)^97^ | No | Yes | Yes | Unclear | Unclear | Yes | Yes | Yes | Yes | No |
| Bellan et al (2021)^98^ | Yes | Yes | Yes | No | No | No | Yes | Yes | No | Yes |
| Rank et al (2021)^99^ | No | Yes | Yes | No | No | No | Yes | Yes | Yes | Yes |
| Chai et al (2021)^100^ | No | Yes | Yes | No | No | No | Yes | Yes | Yes | Yes |
| Tortajada et al (2022)^101^ | Yes | Yes | Yes | Unclear | Unclear | No | Yes | Yes | Yes | Yes |
| Peñas et al (2021)^102^ | No | Yes | No | No | No | Yes | Yes | Yes | Yes | Yes |
| Rizzo et al (2021)^103^ | No | No | No | No | No | No | Yes | Yes | No | No |
| Tessitore et al (2021)^104^ | Yes | Yes | Yes | Unclear | Unclear | No | Yes | Yes | Yes | Yes |
| Yang et al (2022)^105^ | Yes | Yes | No | Unclear | Unclear | Yes | Yes | Yes | Yes | Yes |
| Peluso et al (2021)^106^ | No | Yes | Yes | No | No | Yes | Yes | Yes | Yes | Yes |
| Gutiérrez-Canales et al (2024)^107^ | Yes | Yes | Yes | No | No | No | Yes | Yes | Yes | Yes |
| Petrakis et al (2024)^108^ | Yes | Yes | Yes | No | No | No | Yes | Yes | Yes | Yes |

**Table S2C JBI Critical Appraisal Checklist for analytical cross sectional studies**

|  | Were the criteria for inclusion in the sample clearly defined? | Were the study subjects and the setting described in detail? | Was the exposure measured in a valid and reliable way? | Were objective, standard criteria used for measurement of the condition? | Were confounding factors identified? | Were strategies to deal with confounding factors stated? | Were the outcomes measured in a valid and reliable way? | Was appropriate statistical analysis used? |
| --- | --- | --- | --- | --- | --- | --- | --- | --- |
| Newlands et al (2023)^109^ | No | Yes | Yes | Yes | Yes | No | Yes | No |
| Ahmed et al (2023)^110^ | No | Yes | Yes | Yes | Yes | Yes | No | Yes |
| Shang et al (2024)^111^ | No | Yes | No | No | Yes | Yes | Yes | Yes |
| Daitch et al (2022)^112^ | No | Yes | Yes | Yes | Yes | Yes | Yes | Yes |
| Gasnier et al (2022)^113^ | Yes | Yes | No | No | Yes | Yes | Yes | Yes |
| Morioka et al (2023)^114^ | No | Yes | No | No | Yes | Yes | No | Yes |
| Silva et al (2023)^115^ | No | No | Yes | Yes | Yes | No | No | No |
| Kumar et al (2022)^116^ | Yes | Yes | No | No | No | No | No | No |
| Bougea et al (2023)^117^ | Yes | Yes | No | Yes | Yes | No | Yes | No |
| Leon et al (2022)^118^ | Yes | Yes | Yes | Yes | Yes | Yes | No | Yes |
| Raj et al (2022)^119^ | No | Yes | Yes | Yes | Yes | Yes | No | Yes |
| Tran et al (2023)^120^ | Yes | Yes | Yes | Yes | Yes | No | No | No |
| Oliveira et al (2023)^121^ | Yes | Yes | Yes | Yes | Yes | Yes | Yes | Yes |
| Maamar et al (2022)^122^ | Yes | Yes | Yes | Yes | Yes | Yes | Yes | Yes |
| Ho et al (2023)^123^ | No | Yes | Yes | Yes | Yes | No | Yes | No |
| Nehme et al (2023)^124^ | No | Yes | No | No | Yes | Yes | Yes | Yes |
| Kosowan et al (2023)^125^ | No | Yes | No | No | No | No | No | No |
| Muñiz et al (2021)^126^ | Yes | Yes | No | No | Yes | No | Yes | No |
| Peñas et al (2022)^127^ | No | Yes | Yes | Yes | Yes | Yes | No | Yes |
| Darji et al (2024)^128^ | Yes | Yes | No | No | No | No | No | No |
| Savith et al (2024)^129^ | No | Yes | No | No | Yes | Yes | Yes | Yes |
| Bruijn et al (2024)^130^ | Yes | Yes | No | No | No | No | No | No |
| Catalán et al (2024)^131^ | Yes | Yes | No | No | No | No | No | No |

**Table S3 Subgroup Analysis of Symptom Prevalence by Continent**

**Table S3A 3^rd^** Month Follow-Up

| Symptom | Africa | | | | | | America | | | | | | Asia | | | | | | Europe | | | | | |
| --- | --- | --- | --- | --- | --- | --- | --- | --- | --- | --- | --- | --- | --- | --- | --- | --- | --- | --- | --- | --- | --- | --- | --- | --- |
|  | Cases | Total | Prevalence | CI Lower | CI Upper | I^2^ | Cases | Total | Prevalence | CI Lower | CI Upper | I^2^ | Cases | Total | Prevalence | CI Lower | CI Upper | I^2^ | Cases | Total | Prevalence | CI Lower | CI Upper | I^2^ |
| Chest pain | 138 | 1320 | 10.431 | 8.782 | 12.08 | 0 | 222 | 814 | 26.918 | 12.154 | 41.682 | 94.852 | 420 | 4258 | 9.909 | 6.138 | 13.68 | 96.42 | 571 | 6735 | 14.08 | 8.778 | 19.381 | 97.869 |
| Cough | 89 | 1320 | 9.897 | 1.037 | 18.758 | 77.89 | 235 | 922 | 23.95 | 2.638 | 45.263 | 98.737 | 1914 | 9023 | 20.13 | 11.674 | 28.585 | 99.121 | 819 | 6793 | 26.863 | 13.624 | 40.103 | 99.611 |
| Dyspnea | 494 | 1320 | 56.038 | 14.571 | 97.504 | 98.525 | 446 | 1028 | 45.976 | 26.514 | 65.438 | 97.605 | 2323 | 8937 | 29.184 | 18.109 | 40.26 | 99.382 | 3090 | 12647 | 36.714 | 26.093 | 47.336 | 99.509 |
| Expectoration | 42 | 1320 | 3.179 | 2.233 | 4.126 | 0 | / | / | / | / | / | / | 507 | 4926 | 9.101 | 4.865 | 13.336 | 91.894 | / | / | / | / | / | / |
| Fatigue | 983 | 1320 | 67.514 | 50.29 | 84.737 | 88.523 | 583 | 1189 | 44.199 | 22.482 | 65.916 | 98.727 | 4371 | 9023 | 53.329 | 41.722 | 64.936 | 99.37 | 5943 | 12782 | 51.432 | 41.311 | 61.554 | 99.272 |
| Abdominal stomach pain | 44 | 1249 | 3.523 | / | / | / | / | / | / | / | / | / | 385 | 7674 | 4.202 | 2.673 | 5.732 | 88.128 | 173 | 4787 | 2.814 | 0.425 | 5.203 | 95.774 |
| Abnormal walking posture | / | / | / | / | / | / | 84 | 387 | 21.558 | 17.466 | 25.65 | 0 | 3 | 403 | 0.744 | / | / | / | / | / | / | / | / | / |
| Attention deficit | / | / | / | / | / | / | 358 | 760 | 50.285 | -1.363 | 101.933 | 99.653 | 1590 | 7755 | 19.316 | 10.301 | 28.331 | 99.14 | 1663 | 7210 | 31.327 | 17.924 | 44.731 | 99.243 |
| Memory deficit | / | / | / | / | / | / | 163 | 588 | 15.309 | -5.65 | 36.267 | 98.371 | 1347 | 7876 | 19.583 | 6.925 | 32.24 | 99.702 | 1955 | 11416 | 24.093 | 11.621 | 36.566 | 99.676 |
| Anxiety | / | / | / | / | / | / | 234 | 388 | 60.078 | 54.438 | 65.718 | 9.754 | 558 | 7514 | 19.421 | 4.969 | 33.873 | 99.911 | 79 | 210 | 43.072 | 12.394 | 73.75 | 94.844 |
| Bitterness in the mouth | / | / | / | / | / | / | / | / | / | / | / | / | 7 | 403 | 1.737 | / | / | / | / | / | / | / | / | / |
| Bloating | / | / | / | / | / | / | / | / | / | / | / | / | 6 | 403 | 1.489 | / | / | / | / | / | / | / | / | / |
| Chest tightness | / | / | / | / | / | / | / | / | / | / | / | / | 209 | 1768 | 15.948 | 2.574 | 29.322 | 98.317 | 378 | 4841 | 7.808 | / | / | / |
| Chills | / | / | / | / | / | / | / | / | / | / | / | / | 27 | 403 | 6.7 | / | / | / | / | / | / | / | / | / |
| Constipation | / | / | / | / | / | / | / | / | / | / | / | / | 9 | 403 | 2.233 | / | / | / | 275 | 4360 | 7.342 | 0.296 | 14.389 | 98.517 |
| Decreased appetite | 10 | 1249 | 0.801 | / | / | / | 35 | 268 | 12.682 | 8.489 | 16.874 | 6.448 | 348 | 8159 | 4.002 | 2.677 | 5.328 | 87.383 | 203 | 4731 | 7.073 | -1.591 | 15.738 | 99.558 |
| Depression | / | / | / | / | / | / | 276 | 814 | 35.572 | 15.771 | 55.374 | 96.892 | 486 | 6635 | 12.272 | 4.524 | 20.02 | 98.996 | 470 | 1649 | 32.636 | 20.944 | 44.327 | 89.208 |
| Diarrhea | 38 | 1249 | 3.042 | / | / | / | 38 | 268 | 12.028 | 3.751 | 20.305 | 73.656 | 118 | 2600 | 3.943 | 2.114 | 5.772 | 78.582 | 40 | 643 | 8.956 | 0.466 | 17.445 | 98.983 |
| Dizziness | 116 | 1249 | 9.287 | / | / | / | 242 | 655 | 30.503 | 16.539 | 44.467 | 92.656 | 839 | 8014 | 12.702 | 5.812 | 19.592 | 99.345 | 539 | 5352 | 14.937 | 3.435 | 26.439 | 99.787 |
| Edema | / | / | / | / | / | / | / | / | / | / | / | / | 6 | 660 | 0.867 | 0.16 | 1.574 | 0 | 18 | 4360 | 0.509 | -0.345 | 1.362 | 91.208 |
| Fainting | / | / | / | / | / | / | / | / | / | / | / | / | 1 | 403 | 0.248 | / | / | / | / | / | / | / | / | / |
| Fear of heat | / | / | / | / | / | / | / | / | / | / | / | / | 17 | 403 | 4.218 | / | / | / | / | / | / | / | / | / |
| Fever | 29 | 1249 | 2.322 | / | / | / | 9 | 426 | 2.113 | / | / | / | 229 | 5428 | 6.699 | 2.601 | 10.797 | 96.45 | 117 | 565 | 23.78 | -1.949 | 49.509 | 99.16 |
| Hallucinations | / | / | / | / | / | / | / | / | / | / | / | / | 1 | 403 | 0.248 | / | / | / | / | / | / | / | / | / |
| Headache | 258 | 1249 | 20.657 | / | / | / | 400 | 1189 | 31.609 | 15.757 | 47.461 | 97.437 | 2039 | 8727 | 13.715 | 8.35 | 19.08 | 98.063 | 1070 | 6884 | 22.024 | 13.657 | 30.391 | 98.777 |
| Hearing problems | / | / | / | / | / | / | / | / | / | / | / | / | 11 | 660 | 1.561 | 0.616 | 2.507 | 0 | / | / | / | / | / | / |
| Insomnia | / | / | / | / | / | / | 290 | 814 | 29.26 | -2.378 | 60.898 | 99.109 | 1474 | 7999 | 20.033 | 10.71 | 29.356 | 99.134 | 781 | 5069 | 25.721 | 16.321 | 35.121 | 98.074 |
| Joint pain | 175 | 1249 | 14.011 | / | / | / | 371 | 814 | 50.517 | 32.755 | 68.278 | 95.655 | 162 | 1207 | 12.901 | 0.553 | 25.249 | 98.613 | 873 | 6922 | 15.659 | 8.574 | 22.744 | 98.698 |
| Myalgia | 156 | 1249 | 12.49 | / | / | / | 185 | 588 | 35.552 | 7.45 | 63.654 | 97.975 | 607 | 2370 | 21.733 | 11.903 | 31.562 | 97.351 | 2167 | 11590 | 24.424 | 15.806 | 33.042 | 99.342 |
| Dysmenorrhea | / | / | / | / | / | / | / | / | / | / | / | / | 5 | 403 | 1.241 | / | / | / | / | / | / | / | / | / |
| Nasal congestion | 17 | 1249 | 1.361 | / | / | / | 14 | 108 | 12.963 | / | / | / | 16 | 403 | 3.97 | / | / | / | / | / | / | / | / | / |
| Nausea Vomit | 25 | 1249 | 2.002 | / | / | / | / | / | / | / | / | / | 288 | 5012 | 3.762 | 0.893 | 6.631 | 93.837 | 154 | 1902 | 14.725 | -1.477 | 30.927 | 99.265 |
| Paresthesia | / | / | / | / | / | / | / | / | / | / | / | / | 284 | 5007 | 3.444 | 0.502 | 6.386 | 95.102 | 251 | 4729 | 9.097 | 2.277 | 15.916 | 98.486 |
| Palpitations | / | / | / | / | / | / | 32 | 426 | 7.512 | / | / | / | 493 | 3846 | 10.86 | 6.044 | 15.675 | 95.468 | 716 | 11000 | 11.316 | 2.389 | 20.242 | 99.722 |
| Rhinorrhea | / | / | / | / | / | / | / | / | / | / | / | / | 1349 | 4669 | 18.071 | -7.595 | 43.737 | 99.756 | 8 | 191 | 4.188 | / | / | / |
| Seizures | 2 | 1249 | 0.16 | / | / | / | / | / | / | / | / | / | 1 | 403 | 0.248 | / | / | / | 2 | 1531 | 0.131 | / | / | / |
| Sexual dysfunction | / | / | / | / | / | / | / | / | / | / | / | / | 5 | 403 | 1.241 | / | / | / | / | / | / | / | / | / |
| Skin rash | 35 | 1249 | 2.802 | / | / | / | 8 | 334 | 2.395 | / | / | / | 404 | 7688 | 7.637 | 2.911 | 12.364 | 98.796 | 205 | 4875 | 3.733 | 1.109 | 6.358 | 94.176 |
| Loss of Smell | 44 | 1249 | 3.523 | / | / | / | 82 | 641 | 22.26 | -1.84 | 46.36 | 98.48 | 1143 | 8481 | 9.823 | 5.674 | 13.972 | 98.214 | 1255 | 9147 | 16.684 | 10.302 | 23.065 | 98.209 |
| Loss of Taste | 51 | 1249 | 4.083 | / | / | / | 66 | 587 | 21.553 | -7.819 | 50.925 | 99.033 | 708 | 8125 | 7.731 | 5.35 | 10.111 | 92.347 | 599 | 7593 | 13.434 | 8.856 | 18.011 | 95.655 |
| Sneezing | / | / | / | / | / | / | / | / | / | / | / | / | 839 | 4669 | 10.508 | -7.169 | 28.184 | 99.775 | / | / | / | / | / | / |
| Sore throat | / | / | / | / | / | / | 24 | 496 | 7.902 | -1.869 | 17.673 | 93.911 | 755 | 6121 | 8.138 | 2.404 | 13.873 | 98.305 | 72 | 965 | 9.081 | 2.95 | 15.211 | 95.408 |
| Itchy throat | / | / | / | / | / | / | / | / | / | / | / | / | 20 | 403 | 4.963 | / | / | / | / | / | / | / | / | / |
| Swallowing problems | / | / | / | / | / | / | 73 | 334 | 21.856 | / | / | / | 20 | 533 | 7.1 | -6.965 | 21.164 | 95.32 | 30 | 4360 | 0.855 | -0.471 | 2.182 | 94.108 |
| Sweating | / | / | / | / | / | / | / | / | / | / | / | / | 187 | 2027 | 14.064 | 2.913 | 25.216 | 98.236 | / | / | / | / | / | / |
| Thirst | / | / | / | / | / | / | / | / | / | / | / | / | 13 | 403 | 3.226 | / | / | / | 1 | 236 | 0.424 | / | / | / |
| Tinnitus | / | / | / | / | / | / | / | / | / | / | / | / | 532 | 4782 | 9.511 | 0.391 | 18.631 | 99.227 | 213 | 4360 | 5.554 | 0.898 | 10.21 | 97.391 |
| Twitching | / | / | / | / | / | / | / | / | / | / | / | / | 2 | 403 | 0.496 | / | / | / | / | / | / | / | / | / |
| Urination problems | / | / | / | / | / | / | / | / | / | / | / | / | 12 | 533 | 2.569 | -0.345 | 5.483 | 61.634 | 12 | 4360 | 0.311 | -0.156 | 0.778 | 81.944 |
| Weakness in the limbs | / | / | / | / | / | / | 191 | 334 | 57.186 | / | / | / | 369 | 2043 | 27.172 | -0.234 | 54.578 | 99.519 | 288 | 601 | 38.577 | 11.276 | 65.877 | 97.826 |
| Weight loss | / | / | / | / | / | / | / | / | / | / | / | / | 9 | 403 | 2.233 | / | / | / | 132 | 4551 | 3.344 | 1.623 | 5.065 | 85.33 |
| Hair loss | / | / | / | / | / | / | 197 | 480 | 41.023 | 36.625 | 45.421 | 0 | 234 | 900 | 25.112 | 7.009 | 43.216 | 98.884 | 140 | 1990 | 11.625 | 1.985 | 21.264 | 98.451 |
| Seeing problems | 190 | 1249 | 15.212 | / | / | / | / | / | / | / | / | / | 3 | 257 | 1.167 | / | / | / | 246 | 4538 | 5.184 | 1.48 | 8.888 | 95.341 |
| Cognitive dysfunction | / | / | / | / | / | / | 9 | 53 | 16.981 | / | / | / | 459 | 4535 | 10.105 | 9.228 | 10.982 | 0 | 239 | 4956 | 11.786 | -3.416 | 26.988 | 94.201 |
| Red eye | / | / | / | / | / | / | 5 | 54 | 9.259 | / | / | / | / | / | / | / | / | / | 2 | 191 | 1.047 | / | / | / |
| Earache | / | / | / | / | / | / | / | / | / | / | / | / | 132 | 4266 | 3.094 | / | / | / | / | / | / | / | / | / |
| Menstrual irregularities | / | / | / | / | / | / | / | / | / | / | / | / | 185 | 4266 | 4.337 | / | / | / | / | / | / | / | / | / |
| Sore eyes | / | / | / | / | / | / | / | / | / | / | / | / | 145 | 4266 | 3.399 | / | / | / | / | / | / | / | / | / |
| Tremor | / | / | / | / | / | / | / | / | / | / | / | / | 344 | 4266 | 8.064 | / | / | / | / | / | / | / | / | / |
| Arrhythmia | / | / | / | / | / | / | 16 | 334 | 4.79 | / | / | / | / | / | / | / | / | / | / | / | / | / | / | / |

**Table S3B 6^th^** Month Follow-Up

| Symptom | Africa | | | | | | America | | | | | | Asia | | | | | | Europe | | | | | |
| --- | --- | --- | --- | --- | --- | --- | --- | --- | --- | --- | --- | --- | --- | --- | --- | --- | --- | --- | --- | --- | --- | --- | --- | --- |
|  | Cases | Total | Prevalence | CI Lower | CI Upper | I^2^ | Cases | Total | Prevalence | CI Lower | CI Upper | I^2^ | Cases | Total | Prevalence | CI Lower | CI Upper | I^2^ | Cases | Total | Prevalence | CI Lower | CI Upper | I^2^ |
| Anxiety | / | / | / | / | / | / | 82 | 487 | 16.779 | 13.462 | 20.097 | 0 | 1262 | 5376 | 24.643 | 13.663 | 35.623 | 99.368 | 139 | 558 | 27.613 | 12.782 | 42.445 | 96.114 |
| Cough | 2 | 71 | 2.817 | / | / | / | 106 | 1258 | 7.18 | 3.047 | 11.313 | 89.751 | 1448 | 6688 | 13.726 | 7.651 | 19.801 | 98.183 | 266 | 1849 | 18.018 | 9.272 | 26.765 | 97.725 |
| Depression | / | / | / | / | / | / | 104 | 1125 | 7.969 | 3.143 | 12.795 | 91.66 | 1060 | 4447 | 21.076 | 8.177 | 33.974 | 99.053 | 240 | 1348 | 25.752 | 7.869 | 43.635 | 98.707 |
| Dyspnea | 24 | 71 | 33.803 | / | / | / | 273 | 1167 | 22.78 | 15.516 | 30.044 | 88.758 | 1692 | 6788 | 23.131 | 16.11 | 30.152 | 97.614 | 2095 | 7293 | 39.593 | 31.663 | 47.523 | 97.292 |
| Fatigue | 16 | 71 | 22.535 | / | / | / | 486 | 1308 | 32.815 | 17.501 | 48.128 | 97.913 | 6817 | 9746 | 54.907 | 44.451 | 65.362 | 99.349 | 4410 | 8036 | 48.379 | 40.956 | 55.803 | 97.244 |
| Hair loss | / | / | / | / | / | / | 232 | 1258 | 16.005 | 7.896 | 24.113 | 94.624 | 2328 | 6364 | 25.035 | 15.496 | 34.574 | 98.542 | 441 | 2075 | 18.364 | 7.248 | 29.479 | 99.065 |
| Headache | / | / | / | / | / | / | 211 | 1308 | 17.472 | 10.006 | 24.937 | 94.473 | 590 | 6101 | 8.741 | 3.484 | 13.997 | 99.038 | 405 | 2618 | 19.435 | 12.622 | 26.249 | 97.318 |
| Joint pain | / | / | / | / | / | / | 178 | 1125 | 14.851 | 10.87 | 18.832 | 72.254 | 805 | 4246 | 21.33 | 14.093 | 28.567 | 97.312 | 340 | 1301 | 18.384 | 10.43 | 26.338 | 93.4 |
| Loss of Smell | / | / | / | / | / | / | 84 | 698 | 18.408 | 4.373 | 32.442 | 96.436 | 935 | 6296 | 12.758 | 7.304 | 18.212 | 98.233 | 675 | 6684 | 17.991 | 9.321 | 26.661 | 98.574 |
| Chest pain | 2 | 71 | 2.817 | / | / | / | 107 | 1125 | 7.461 | 0.093 | 14.829 | 97.829 | 537 | 5277 | 9.934 | 5.559 | 14.309 | 96.8 | 263 | 2052 | 17.737 | 8.915 | 26.56 | 98.409 |
| Decreased appetite | / | / | / | / | / | / | 8 | 151 | 5.298 | / | / | / | 368 | 4236 | 7.181 | 3.761 | 10.601 | 95.283 | 181 | 1370 | 9.446 | 3.19 | 15.703 | 95.073 |
| Diarrhea | / | / | / | / | / | / | 7 | 193 | 3.329 | 0.173 | 6.485 | 13.344 | 92 | 1712 | 4.116 | 1.062 | 7.17 | 88.805 | 112 | 1444 | 11.38 | 2.527 | 20.232 | 98.893 |
| Dizziness | / | / | / | / | / | / | 35 | 537 | 5.592 | 3.654 | 7.53 | 0.51 | 357 | 5087 | 6.968 | 4.433 | 9.503 | 93.901 | 387 | 1625 | 19.119 | 8.964 | 29.275 | 98.344 |
| Fever | / | / | / | / | / | / | 2 | 406 | 0.303 | -0.231 | 0.837 | 0 | 12 | 1880 | 0.858 | -0.033 | 1.749 | 54.578 | 114 | 1249 | 17.91 | 0.458 | 35.362 | 99.744 |
| Insomnia | / | / | / | / | / | / | 94 | 806 | 9.697 | 0.849 | 18.546 | 96.068 | 2664 | 9476 | 23.272 | 15.811 | 30.734 | 98.859 | 672 | 2086 | 27.053 | 18.483 | 35.623 | 95.691 |
| Myalgia | / | / | / | / | / | / | 165 | 880 | 17.158 | 11.248 | 23.068 | 81.26 | 2750 | 7824 | 20.729 | 7.167 | 34.291 | 99.775 | 1229 | 6173 | 21.929 | 13.326 | 30.531 | 98.325 |
| Palpitations | / | / | / | / | / | / | 56 | 700 | 8.488 | 4.699 | 12.278 | 65.898 | 987 | 7264 | 10.869 | 5.69 | 16.048 | 97.77 | 477 | 5771 | 22.968 | 9.377 | 36.559 | 98.546 |
| Skin rash | / | / | / | / | / | / | / | / | / | / | / | / | 198 | 4655 | 4.23 | 2.623 | 5.837 | 86.087 | 70 | 1376 | 12.229 | -7.196 | 31.653 | 99.782 |
| Loss of Taste | / | / | / | / | / | / | 68 | 883 | 12.533 | -0.39 | 25.456 | 98.672 | 631 | 6296 | 8.358 | 4.674 | 12.042 | 96.88 | 391 | 6588 | 16.15 | 7.416 | 24.884 | 98.886 |
| Sore throat | / | / | / | / | / | / | 22 | 284 | 7.328 | 4.305 | 10.352 | 0 | 156 | 3128 | 6.032 | 2.971 | 9.093 | 92.779 | 121 | 1272 | 15.039 | -0.548 | 30.627 | 99.273 |
| Abdominal pain | / | / | / | / | / | / | 7 | 274 | 2.555 | / | / | / | 12 | 1290 | 0.863 | 0.359 | 1.368 | 0 | 70 | 796 | 14.652 | -2.005 | 31.31 | 98.963 |
| Chills | / | / | / | / | / | / | / | / | / | / | / | / | 1 | 72 | 1.389 | / | / | / | 35 | 441 | 7.776 | 5.279 | 10.273 | 0 |
| Earache | / | / | / | / | / | / | 6 | 274 | 2.19 | / | / | / | / | / | / | / | / | / | 22 | 345 | 6.377 | / | / | / |
| Hoarse voice | / | / | / | / | / | / | 27 | 274 | 9.854 | / | / | / | / | / | / | / | / | / | 4 | 345 | 1.159 | / | / | / |
| Sore eyes | / | / | / | / | / | / | / | / | / | / | / | / | / | / | / | / | / | / | 31 | 345 | 8.986 | / | / | / |
| Red eye | / | / | / | / | / | / | 4 | 151 | 2.649 | / | / | / | 6 | 277 | 2.166 | / | / | / | 2 | 134 | 1.493 | / | / | / |
| Rhinorrhea | / | / | / | / | / | / | / | / | / | / | / | / | 1 | 277 | 0.361 | / | / | / | 47 | 294 | 15.104 | -0.241 | 30.448 | 93.37 |
| Edema | / | / | / | / | / | / | 15 | 274 | 5.474 | / | / | / | 23 | 100 | 23 | / | / | / | / | / | / | / | / | / |
| Fainting | / | / | / | / | / | / | / | / | / | / | / | / | 1 | 100 | 1 | / | / | / | / | / | / | / | / | / |
| Arrhythmia | / | / | / | / | / | / | 30 | 274 | 10.949 | / | / | / | 276 | 1521 | 13.142 | -0.967 | 27.251 | 98.404 | 4 | 107 | 3.738 | / | / | / |
| Attention deficit | / | / | / | / | / | / | 97 | 742 | 10.31 | 4.69 | 15.931 | 84.255 | 1076 | 4560 | 18.751 | 7.914 | 29.589 | 99.144 | 1248 | 5892 | 29.323 | 13.464 | 45.183 | 99.251 |
| Memory deficit | / | / | / | / | / | / | 257 | 833 | 25.754 | 10.935 | 40.573 | 96.328 | 527 | 2666 | 21.12 | 9.361 | 32.879 | 98.616 | 951 | 5620 | 18.373 | 2.595 | 34.151 | 99.097 |
| Cognitive dysfunction | / | / | / | / | / | / | 14 | 50 | 28 | / | / | / | 509 | 2987 | 26.355 | 14.043 | 38.666 | 87.581 | 348 | 5718 | 6.594 | 0.608 | 12.579 | 97.275 |
| Expectoration | / | / | / | / | / | / | 7 | 151 | 4.636 | / | / | / | 19 | 241 | 10.164 | -3.995 | 24.324 | 89.27 | 14 | 112 | 12.5 | / | / | / |
| Nausea Vomit | / | / | / | / | / | / | / | / | / | / | / | / | 163 | 2499 | 5.921 | -0.39 | 12.233 | 98.51 | 57 | 574 | 15.527 | -1.396 | 32.45 | 99.173 |
| Paraesthesia | / | / | / | / | / | / | 73 | 274 | 26.642 | / | / | / | 57 | 923 | 7.957 | -0.336 | 16.25 | 95.801 | 16 | 392 | 3.562 | 0.987 | 6.137 | 44.491 |
| Tinnitus | / | / | / | / | / | / | 5 | 274 | 1.825 | / | / | / | 11 | 168 | 6.664 | -3.469 | 16.798 | 84.042 | 23 | 160 | 14.375 | / | / | / |
| Weight loss | / | / | / | / | / | / | / | / | / | / | / | / | 262 | 819 | 31.99 | / | / | / | 30 | 246 | 12.682 | -5.523 | 30.888 | 94.745 |
| Constipation | / | / | / | / | / | / | 6 | 274 | 2.19 | / | / | / | / | / | / | / | / | / | / | / | / | / | / | / |
| Tremor | / | / | / | / | / | / | 22 | 274 | 8.029 | / | / | / | / | / | / | / | / | / | 18 | 203 | 8.895 | -7.522 | 25.311 | 94.297 |
| Weakness in the limbs | / | / | / | / | / | / | 42 | 610 | 6.772 | 4.346 | 9.198 | 29.182 | 437 | 819 | 53.358 | / | / | / | 329 | 800 | 34.038 | 18.936 | 49.14 | 94.249 |
| Chest tightness | / | / | / | / | / | / | / | / | / | / | / | / | 106 | 1013 | 18.58 | -5.994 | 43.155 | 97.864 | 354 | 4867 | 8.452 | 4.694 | 12.21 | 62.256 |
| Seeing problems | / | / | / | / | / | / | / | / | / | / | / | / | 407 | 3914 | 7.69 | -1.632 | 17.012 | 99.352 | 19 | 243 | 5.988 | -3.845 | 15.821 | 92.395 |
| Sweating | / | / | / | / | / | / | 2 | 336 | 0.595 | -0.227 | 1.418 | 0 | 64 | 1183 | 10.706 | -6.524 | 27.935 | 99.296 | 29 | 160 | 18.125 | / | / | / |
| Swallowing problems | / | / | / | / | / | / | / | / | / | / | / | / | / | / | / | / | / | / | 3 | 112 | 2.679 | / | / | / |
| Hearing problems | / | / | / | / | / | / | / | / | / | / | / | / | 83 | 988 | 5.262 | -3.97 | 14.494 | 98.368 | / | / | / | / | / | / |
| Sexual dysfunction | / | / | / | / | / | / | / | / | / | / | / | / | 223 | 819 | 27.228 | / | / | / | / | / | / | / | / | / |
| Nasal congestion | / | / | / | / | / | / | 12 | 91 | 13.187 | / | / | / | / | / | / | / | / | / | / | / | / | / | / | / |
| Abnormal walking posture | / | / | / | / | / | / | 12 | 50 | 24 | / | / | / | / | / | / | / | / | / | 7 | 107 | 6.542 | / | / | / |
| Skin lesion | / | / | / | / | / | / | / | / | / | / | / | / | 3 | 169 | 1.775 | / | / | / | 10 | 83 | 12.048 | / | / | / |

**Table S3C 12^th^** Month Follow-Up

| Symptom | Africa | | | | | | America | | | | | | Asia | | | | | | Europe | | | | | |
| --- | --- | --- | --- | --- | --- | --- | --- | --- | --- | --- | --- | --- | --- | --- | --- | --- | --- | --- | --- | --- | --- | --- | --- | --- |
|  | Cases | Total | Prevalence | CI Lower | CI Upper | I^2^ | Cases | Total | Prevalence | CI Lower | CI Upper | I^2^ | Cases | Total | Prevalence | CI Lower | CI Upper | I^2^ | Cases | Total | Prevalence | CI Lower | CI Upper | I^2^ |
| Anxiety | / | / | / | / | / | / | 92 | 495 | 22.541 | 5.491 | 39.59 | 97.215 | 720 | 3007 | 27.189 | 16.276 | 38.101 | 97.761 | 386 | 1731 | 26.589 | 12.614 | 40.563 | 98.427 |
| Cough | 1 | 71 | 1.408 | / | / | / | 30 | 476 | 5.925 | 2.244 | 9.605 | 69.29 | 308 | 3192 | 7.566 | 4.071 | 11.062 | 92.364 | 684 | 4894 | 14.556 | 9.963 | 19.149 | 95.725 |
| Depression | / | / | / | / | / | / | 27 | 407 | 8.732 | -3.406 | 20.87 | 97.917 | 109 | 391 | 30.346 | 11.704 | 48.988 | 95.068 | 298 | 1777 | 25.434 | 8.398 | 42.469 | 99.11 |
| Diarrhea | / | / | / | / | / | / | / | / | / | / | / | / | 50 | 2869 | 1.643 | 1.178 | 2.108 | 0 | 147 | 1098 | 9.316 | 2.31 | 16.322 | 94.997 |
| Dyspnea | 16 | 71 | 22.535 | / | / | / | 135 | 495 | 29.518 | 13.068 | 45.967 | 95.458 | 427 | 3106 | 12.084 | 7.427 | 16.741 | 92.928 | 3445 | 10943 | 34.16 | 27.539 | 40.782 | 98.156 |
| Fatigue | 8 | 71 | 11.268 | / | / | / | 234 | 612 | 39.184 | 20.279 | 58.09 | 97.484 | 2318 | 4484 | 39.581 | 30.004 | 49.158 | 97.669 | 6899 | 11740 | 53.423 | 46.421 | 60.425 | 98.242 |
| Hair loss | / | / | / | / | / | / | 59 | 341 | 17.205 | 13.203 | 21.208 | 0 | 326 | 1625 | 16.935 | 11.971 | 21.899 | 82.442 | 620 | 2869 | 25.784 | 10.402 | 41.166 | 99.344 |
| Headache | / | / | / | / | / | / | 86 | 612 | 16.64 | 5.993 | 27.287 | 96.337 | 272 | 4109 | 6.324 | 3.823 | 8.825 | 90.039 | 967 | 3860 | 22.437 | 15.69 | 29.184 | 97.005 |
| Joint pain | / | / | / | / | / | / | 52 | 272 | 25.346 | -5.258 | 55.95 | 98.178 | 379 | 1661 | 21.064 | 14.832 | 27.296 | 88.17 | 797 | 2608 | 21.336 | 7.562 | 35.11 | 98.999 |
| Loss of Smell | / | / | / | / | / | / | 31 | 169 | 19.377 | -5.166 | 43.921 | 96.323 | 243 | 4239 | 8.267 | 3.532 | 13.003 | 98.065 | 740 | 8154 | 12.777 | 8.201 | 17.354 | 97.463 |
| Chest pain | 1 | 71 | 1.408 | / | / | / | 15 | 272 | 7.891 | -6.718 | 22.5 | 98.992 | 206 | 1550 | 10.457 | 5.029 | 15.885 | 90.868 | 586 | 5365 | 10.029 | 6.615 | 13.443 | 95.105 |
| Arrhythmia | / | / | / | / | / | / | 2 | 110 | 1.818 | / | / | / | 35 | 179 | 19.216 | 13.461 | 24.97 | 0 | 438 | 804 | 41.963 | 14.132 | 69.794 | 99.182 |
| Attention deficit | / | / | / | / | / | / | 35 | 308 | 12.458 | -8.866 | 33.782 | 99.147 | 35 | 179 | 19.216 | 13.461 | 24.97 | 0 | 1568 | 6423 | 41.809 | 28.805 | 54.812 | 98.926 |
| Memory deficit | / | / | / | / | / | / | 93 | 476 | 19.936 | 9.614 | 30.258 | 89.878 | 101 | 400 | 30.614 | 7.214 | 54.014 | 97.638 | 1488 | 6841 | 31.135 | 17.418 | 44.852 | 99.454 |
| Cognitive dysfunction | / | / | / | / | / | / | 16 | 48 | 33.333 | / | / | / | 36 | 216 | 16.126 | 1.316 | 30.936 | 90.937 | 640 | 6273 | 23.746 | 8.508 | 38.984 | 99.561 |
| Decreased appetite | / | / | / | / | / | / | 4 | 52 | 7.692 | / | / | / | 123 | 3879 | 3.41 | 1.827 | 4.993 | 87.351 | 206 | 2376 | 6.564 | 1.779 | 11.349 | 96.34 |
| Dizziness | / | / | / | / | / | / | 30 | 320 | 10.888 | 2.365 | 19.411 | 87.227 | 364 | 4109 | 8.755 | 7.434 | 10.075 | 44.271 | 498 | 1412 | 20.048 | 5.143 | 34.953 | 98.526 |
| Expectoration | / | / | / | / | / | / | / | / | / | / | / | / | 217 | 2970 | 7.033 | 6.115 | 7.951 | 0 | 13 | 223 | 4.778 | -3.029 | 12.585 | 88.328 |
| Insomnia | / | / | / | / | / | / | 26 | 272 | 14.546 | -11.121 | 40.213 | 99.384 | 559 | 1882 | 25.027 | 17.565 | 32.49 | 93.198 | 1539 | 4210 | 33.282 | 23.326 | 43.238 | 98.232 |
| Myalgia | / | / | / | / | / | / | 75 | 344 | 24.18 | 9.406 | 38.954 | 91.749 | 623 | 4379 | 10.94 | 7.039 | 14.841 | 93.947 | 1986 | 7561 | 24.954 | 13.164 | 36.744 | 99.286 |
| Paresthesia | / | / | / | / | / | / | / | / | / | / | / | / | 33 | 317 | 11.17 | 1.487 | 20.853 | 88.421 | 467 | 1490 | 20.508 | 6.794 | 34.223 | 98.127 |
| Palpitations | / | / | / | / | / | / | 14 | 220 | 6.364 | 3.138 | 9.589 | 0 | 522 | 4348 | 9.731 | 6.01 | 13.452 | 94.309 | 839 | 7456 | 17.035 | 9.314 | 24.756 | 98.669 |
| Skin rash | / | / | / | / | / | / | / | / | / | / | / | / | 123 | 1554 | 6.534 | 4.52 | 8.549 | 48.874 | 214 | 2313 | 7.941 | 0.452 | 15.43 | 98.58 |
| Loss of Taste | / | / | / | / | / | / | 21 | 117 | 20.954 | -18.44 | 60.349 | 96.741 | 179 | 4101 | 4.11 | 2.695 | 5.526 | 77.579 | 574 | 8147 | 11.462 | 6.315 | 16.609 | 98.483 |
| Sore throat | / | / | / | / | / | / | 16 | 121 | 12.172 | 2.695 | 21.65 | 63.351 | 111 | 3376 | 3.758 | 1.964 | 5.551 | 86.705 | 110 | 1287 | 5.943 | 1.933 | 9.953 | 88.994 |
| Tinnitus | / | / | / | / | / | / | / | / | / | / | / | / | 20 | 145 | 13.77 | 8.162 | 19.377 | 0 | / | / | / | / | / | / |
| Abdominal pain | / | / | / | / | / | / | / | / | / | / | / | / | / | / | / | / | / | / | 121 | 669 | 11.412 | -6.955 | 29.779 | 98.587 |
| Chest tightness | / | / | / | / | / | / | / | / | / | / | / | / | 859 | 3000 | 25.039 | 19.661 | 30.417 | 89.467 | 544 | 5051 | 21.1 | -5.252 | 47.451 | 99.431 |
| Constipation | / | / | / | / | / | / | 3 | 52 | 5.769 | / | / | / | / | / | / | / | / | / | 141 | 572 | 24.65 | / | / | / |
| Edema | / | / | / | / | / | / | / | / | / | / | / | / | 90 | 2786 | 2.479 | 1.186 | 3.772 | 78.024 | 293 | 1098 | 26.109 | 0.945 | 51.273 | 99.037 |
| Fainting | / | / | / | / | / | / | / | / | / | / | / | / | / | / | / | / | / | / | 31 | 1098 | 2.808 | 1.831 | 3.785 | 0 |
| Seeing problems | / | / | / | / | / | / | / | / | / | / | / | / | 26 | 117 | 22.222 | / | / | / | 115 | 572 | 20.105 | / | / | / |
| Nausea Vomit | / | / | / | / | / | / | / | / | / | / | / | / | 31 | 3734 | 0.772 | 0.165 | 1.379 | 84.368 | 73 | 724 | 5.093 | -2.127 | 12.314 | 95.391 |
| Weakness in the limbs | / | / | / | / | / | / | 20 | 355 | 5.625 | 3.229 | 8.022 | 0 | / | / | / | / | / | / | 698 | 1686 | 33.831 | 14.008 | 53.655 | 99.016 |
| Weight loss | / | / | / | / | / | / | / | / | / | / | / | / | / | / | / | / | / | / | 57 | 627 | 5.911 | -1.899 | 13.721 | 92.464 |
| Red eye | / | / | / | / | / | / | 8 | 52 | 15.385 | / | / | / | / | / | / | / | / | / | 1 | 97 | 1.031 | / | / | / |
| Rhinorrhea | / | / | / | / | / | / | / | / | / | / | / | / | 2 | 83 | 2.41 | / | / | / | 61 | 598 | 8.989 | 0.571 | 17.407 | 94.938 |
| Arthritis | / | / | / | / | / | / | / | / | / | / | / | / | / | / | / | / | / | / | 12 | 68 | 17.647 | / | / | / |
| Hearing problems | / | / | / | / | / | / | / | / | / | / | / | / | 3 | 255 | 0.987 | -0.225 | 2.199 | 0 | 54 | 496 | 9.597 | 0.187 | 19.006 | 94.158 |
| Swallowing problems | / | / | / | / | / | / | / | / | / | / | / | / | 10 | 83 | 12.048 | / | / | / | 22 | 410 | 5.259 | 3.1 | 7.419 | 0 |
| Skin lesions | / | / | / | / | / | / | / | / | / | / | / | / | / | / | / | / | / | / | 43 | 609 | 5.968 | 2.122 | 9.815 | 65.246 |
| Sweating | / | / | / | / | / | / | / | / | / | / | / | / | 1002 | 2786 | 24.922 | 7.554 | 42.29 | 99.462 | 134 | 526 | 25.475 | / | / | / |
| Nasal congestion | / | / | / | / | / | / | 20 | 69 | 28.986 | / | / | / | 6 | 1725 | 0.346 | 0.069 | 0.623 | 0 | / | / | / | / | / | / |
| Abnormal walking posture | / | / | / | / | / | / | 13 | 48 | 27.083 | / | / | / | / | / | / | / | / | / | 3 | 86 | 3.488 | / | / | / |
| Chills | / | / | / | / | / | / | / | / | / | / | / | / | 11 | 2648 | 0.254 | -0.05 | 0.558 | 53.441 | / | / | / | / | / | / |
| Fever | / | / | / | / | / | / | / | / | / | / | / | / | 1 | 117 | 0.855 | / | / | / | 78 | 682 | 6.237 | -3.507 | 15.98 | 97.89 |
| Sexual dysfunction | / | / | / | / | / | / | / | / | / | / | / | / | / | / | / | / | / | / | 1 | 86 | 1.163 | / | / | / |
| Twitching | / | / | / | / | / | / | / | / | / | / | / | / | / | / | / | / | / | / | 1 | 86 | 1.163 | / | / | / |
| Tremor | / | / | / | / | / | / | / | / | / | / | / | / | / | / | / | / | / | / | 2 | 132 | 1.515 | / | / | / |
| Sore eyes | / | / | / | / | / | / | / | / | / | / | / | / | / | / | / | / | / | / | 12 | 83 | 14.458 | / | / | / |

**Table S3D 24^th^** Month Follow-Up

| Symptom | America | | | | | | Asia | | | | | | Europe | | | | | |
| --- | --- | --- | --- | --- | --- | --- | --- | --- | --- | --- | --- | --- | --- | --- | --- | --- | --- | --- |
|  | Cases | Total | Prevalence | CI Lower | CI Upper | I^2^ | Cases | Total | Prevalence | CI Lower | CI Upper | I^2^ | Cases | Total | Prevalence | CI Lower | CI Upper | I^2^ |
| Arrhythmia | / | / | / | / | / | / | 4 | 75 | 5.333 | / | / | / | 8 | 423 | 1.891 | / | / | / |
| Attention defcit | 18 | 40 | 45 | / | / | / | 23 | 75 | 30.667 | / | / | / | 466 | 1275 | 40.647 | 17.356 | 63.939 | 99.003 |
| Memory defcit | 16 | 40 | 40 | / | / | / | 46 | 195 | 25.706 | 3.839 | 47.574 | 91.616 | 595 | 1275 | 50.449 | 33.207 | 67.69 | 97.514 |
| Anxiety | 14 | 40 | 35 | / | / | / | 194 | 824 | 21.089 | 7.854 | 34.325 | 96.455 | 98 | 349 | 30.673 | 3.764 | 57.582 | 96.746 |
| Chills | / | / | / | / | / | / | 3 | 704 | 0.336 | -0.091 | 0.764 | 0 | 12 | 210 | 5.714 | / | / | / |
| Cognitive dysfunction | / | / | / | / | / | / | 6 | 75 | 8 | / | / | / | 45 | 423 | 10.638 | / | / | / |
| Cough | / | / | / | / | / | / | 107 | 824 | 16.061 | 5.11 | 27.012 | 95.26 | 79 | 293 | 22.507 | 2.115 | 42.899 | 94.625 |
| Decreased appetite | / | / | / | / | / | / | 51 | 1474 | 3.261 | 0.881 | 5.641 | 87.815 | 19 | 210 | 9.048 | / | / | / |
| Depression | / | / | / | / | / | / | 19 | 75 | 25.333 | / | / | / | 127 | 349 | 38.044 | 19.984 | 56.105 | 91.833 |
| Diarrhea | / | / | / | / | / | / | 27 | 824 | 2.836 | 1.704 | 3.968 | 0.126 | 52 | 633 | 11.471 | -8.602 | 31.544 | 97.98 |
| Dizziness | / | / | / | / | / | / | 187 | 1474 | 12.229 | 4.189 | 20.269 | 97.051 | 297 | 852 | 32.709 | 22.121 | 43.296 | 90.036 |
| Dyspnea | 23 | 40 | 57.5 | / | / | / | 73 | 824 | 7.072 | 2.445 | 11.699 | 86.643 | 375 | 1219 | 28.578 | 9.157 | 47.999 | 98.543 |
| Expectoration | / | / | / | / | / | / | 66 | 704 | 12.271 | 2.639 | 21.903 | 93.775 | / | / | / | / | / | / |
| Fatigue | 21 | 40 | 52.5 | / | / | / | 724 | 1474 | 43.91 | 33.837 | 53.983 | 92.823 | 909 | 1358 | 65.501 | 52.311 | 78.691 | 96.281 |
| Fever | / | / | / | / | / | / | 10 | 195 | 4.451 | 0.554 | 8.348 | 45.963 | 12 | 210 | 5.714 | / | / | / |
| Hair loss | / | / | / | / | / | / | 203 | 1474 | 13.628 | 4.142 | 23.114 | 98.246 | 100 | 716 | 13.928 | 11.393 | 16.463 | 0 |
| Headache | / | / | / | / | / | / | 114 | 1474 | 6.667 | 2.614 | 10.72 | 90.872 | 175 | 432 | 34.349 | 10.267 | 58.431 | 96.996 |
| Insomnia | / | / | / | / | / | / | 332 | 845 | 28.571 | 7.289 | 49.854 | 97.575 | 276 | 852 | 34.123 | 24.953 | 43.293 | 85.65 |
| Joint pain | / | / | / | / | / | / | 134 | 845 | 12.931 | 0.289 | 25.573 | 97.766 | 170 | 503 | 33.797 | / | / | / |
| Myalgia | / | / | / | / | / | / | 166 | 1474 | 9.774 | 5.081 | 14.467 | 90.076 | 93 | 139 | 66.906 | / | / | / |
| Nausea Vomit | / | / | / | / | / | / | 44 | 1474 | 2.606 | 1.144 | 4.069 | 65.374 | 13 | 210 | 6.19 | / | / | / |
| Paresthesia | / | / | / | / | / | / | 14 | 195 | 8.256 | -5.745 | 22.257 | 90.613 | 45 | 210 | 21.429 | / | / | / |
| Palpitations | / | / | / | / | / | / | 190 | 1474 | 11.743 | 2.735 | 20.75 | 98.692 | 55 | 210 | 26.19 | / | / | / |
| Rhinorrhea | / | / | / | / | / | / | 11 | 75 | 14.667 | / | / | / | 83 | 210 | 39.524 | / | / | / |
| Skin rash | / | / | / | / | / | / | 39 | 845 | 3.506 | 0.291 | 6.72 | 82.86 | 38 | 633 | 6.86 | 0.322 | 13.398 | 88.112 |
| Loss of Smell | / | / | / | / | / | / | 84 | 1474 | 4.392 | 0.114 | 8.67 | 96.422 | 79 | 633 | 15.122 | -1.487 | 31.732 | 96.469 |
| Loss of Taste | / | / | / | / | / | / | 40 | 1474 | 1.753 | -0.216 | 3.721 | 93.404 | 45 | 633 | 9.303 | -4.707 | 23.313 | 96.5 |
| Sore throat | / | / | / | / | / | / | 80 | 1474 | 4.474 | -0.275 | 9.223 | 97.761 | 53 | 633 | 10.358 | -2.496 | 23.212 | 95.544 |
| Tinnitus | / | / | / | / | / | / | 9 | 75 | 12 | / | / | / | / | / | / | / | / | / |
| Chest pain | / | / | / | / | / | / | 87 | 770 | 8.103 | -1.144 | 17.349 | 95.06 | 72 | 293 | 18.896 | -6.466 | 44.259 | 97.437 |
| Chest tightness | / | / | / | / | / | / | 86 | 749 | 9.054 | 1.095 | 17.013 | 94.643 | / | / | / | / | / | / |
| Edema | / | / | / | / | / | / | 12 | 629 | 1.851 | 0.798 | 2.904 | 0 | / | / | / | / | / | / |
| Sweating | / | / | / | / | / | / | 31 | 629 | 4.863 | 3.183 | 6.544 | 0 | / | / | / | / | / | / |
| Seeing problems | / | / | / | / | / | / | / | / | / | / | / | / | 126 | 855 | 17.777 | 2.859 | 32.695 | 97.346 |
| Hoarse voice | / | / | / | / | / | / | / | / | / | / | / | / | 6 | 423 | 1.418 | / | / | / |
| Skin lesion | / | / | / | / | / | / | / | / | / | / | / | / | 6 | 83 | 7.229 | / | / | / |
| Abdominal stomach pain | / | / | / | / | / | / | / | / | / | / | / | / | 31 | 210 | 14.762 | / | / | / |
| Abnormal walking posture | / | / | / | / | / | / | / | / | / | / | / | / | 82 | 349 | 25.991 | -0.182 | 52.163 | 96.841 |
| Bloating | / | / | / | / | / | / | / | / | / | / | / | / | 94 | 210 | 44.762 | / | / | / |
| Nasal congestion | / | / | / | / | / | / | / | / | / | / | / | / | 80 | 210 | 38.095 | / | / | / |
| Red eye | / | / | / | / | / | / | / | / | / | / | / | / | 204 | 713 | 22.334 | -7.488 | 52.156 | 99.155 |
| Swallowing problems | / | / | / | / | / | / | / | / | / | / | / | / | 75 | 349 | 21.404 | 16.705 | 26.102 | 14.753 |
| Weakness in the limbs | / | / | / | / | / | / | / | / | / | / | / | / | 330 | 852 | 42.569 | 15.294 | 69.844 | 98.572 |
| Discoloration of toes and fingers | / | / | / | / | / | / | / | / | / | / | / | / | 4 | 210 | 1.905 | / | / | / |
| Hearing problems | / | / | / | / | / | / | / | / | / | / | / | / | 43 | 139 | 30.935 | / | / | / |

**Table S4 Correlations Network Analysis Results of Symptoms**

**Table S4A 3^rd^ Month Follow-Up**

| AverageShortestPathLength | BetweennessCentrality | ClosenessCentrality | ClusteringCoefficient | Degree | Eccentricity | IsSingleNode | name | NeighborhoodConnectivity | NumberOfDirectedEdges | NumberOfUndirectedEdges | PartnerOfMultiEdgedNodePairs | Radiality | selected | SelfLoops | shared name | Stress | TopologicalCoefficient |
| --- | --- | --- | --- | --- | --- | --- | --- | --- | --- | --- | --- | --- | --- | --- | --- | --- | --- |
| 1.3428571428571427 | 0.018313621591695074 | 0.7446808510638299 | 0.7509881422924901 | 23 | 2 | FALSE | Chest pain | 21.869565217391305 | 0 | 23 | 0 | 0.9885714285714287 | TRUE | 0 | Chest pain | 174 | 0.6248447204968944 |
| 1.3714285714285714 | 0.036157470290197995 | 0.7291666666666666 | 0.7747035573122529 | 23 | 3 | FALSE | Loss of Taste | 22.304347826086957 | 0 | 23 | 0 | 0.9876190476190476 | TRUE | 0 | Loss of Taste | 270 | 0.6560102301790282 |
| 1.1428571428571428 | 0.07365722222054977 | 0.875 | 0.6275862068965518 | 30 | 2 | FALSE | Cough | 19.833333333333332 | 0 | 30 | 0 | 0.9952380952380953 | TRUE | 0 | Cough | 424 | 0.5666666666666667 |
| 1.6571428571428573 | 0.004034280378818193 | 0.603448275862069 | 0.8076923076923077 | 13 | 3 | FALSE | Depression | 23.23076923076923 | 0 | 13 | 0 | 0.978095238095238 | TRUE | 0 | Depression | 42 | 0.6832579185520362 |
| 1.2 | 0.034356476281148345 | 0.8333333333333334 | 0.6984126984126984 | 28 | 2 | FALSE | Dizziness | 20.928571428571427 | 0 | 28 | 0 | 0.9933333333333334 | TRUE | 0 | Dizziness | 318 | 0.5979591836734693 |
| 1.4 | 0.01066390601294784 | 0.7142857142857143 | 0.8095238095238095 | 22 | 3 | FALSE | Myalgia | 22.772727272727273 | 0 | 22 | 0 | 0.9866666666666667 | TRUE | 0 | Myalgia | 112 | 0.6697860962566845 |
| 2.0285714285714285 | 9.886307464162135E-5 | 0.49295774647887325 | 0.8333333333333334 | 4 | 3 | FALSE | Hair loss | 25 | 0 | 4 | 0 | 0.9657142857142857 | TRUE | 0 | Hair loss | 2 | 0.8333333333333334 |
| 1.2 | 0.04200856742063446 | 0.8333333333333334 | 0.6798941798941799 | 28 | 2 | FALSE | Headache | 20.642857142857142 | 0 | 28 | 0 | 0.9933333333333334 | TRUE | 0 | Headache | 330 | 0.5897959183673469 |
| 1.4857142857142858 | 0.009532003084634663 | 0.673076923076923 | 0.8596491228070176 | 19 | 3 | FALSE | Sore throat | 23.36842105263158 | 0 | 19 | 0 | 0.9838095238095238 | TRUE | 0 | Sore throat | 76 | 0.6873065015479877 |
| 1.2 | 0.039264388163850174 | 0.8333333333333334 | 0.6576354679802956 | 29 | 3 | FALSE | Fatigue | 20.24137931034483 | 0 | 29 | 0 | 0.9933333333333334 | TRUE | 0 | Fatigue | 322 | 0.5953346855983773 |
| 1.4285714285714286 | 0.007425915107576007 | 0.7 | 0.8428571428571429 | 21 | 3 | FALSE | Loss of Smell | 23.238095238095237 | 0 | 21 | 0 | 0.9857142857142858 | TRUE | 0 | Loss of Smell | 66 | 0.6834733893557423 |
| 1.3142857142857143 | 0.030359300180920513 | 0.7608695652173914 | 0.75 | 25 | 3 | FALSE | Skin rash | 21.72 | 0 | 25 | 0 | 0.9895238095238096 | TRUE | 0 | Skin rash | 198 | 0.6388235294117647 |
| 1.2857142857142858 | 0.030836128062332515 | 0.7777777777777777 | 0.7133333333333334 | 25 | 2 | FALSE | Joint pain | 21.36 | 0 | 25 | 0 | 0.9904761904761905 | TRUE | 0 | Joint pain | 258 | 0.6102857142857143 |
| 1.3428571428571427 | 0.007309258792754401 | 0.7446808510638299 | 0.8115942028985508 | 24 | 3 | FALSE | Decreased appetite | 22.5 | 0 | 24 | 0 | 0.9885714285714287 | TRUE | 0 | Decreased appetite | 112 | 0.6617647058823529 |
| 1.3428571428571427 | 0.014800077278382574 | 0.7446808510638299 | 0.7984189723320159 | 23 | 2 | FALSE | Palpitations | 22.52173913043478 | 0 | 23 | 0 | 0.9885714285714287 | TRUE | 0 | Palpitations | 162 | 0.6434782608695652 |
| 1.2571428571428571 | 0.029691981112451516 | 0.7954545454545455 | 0.7008547008547008 | 27 | 3 | FALSE | Memory deficit | 21 | 0 | 27 | 0 | 0.9914285714285714 | TRUE | 0 | Memory deficit | 254 | 0.6176470588235294 |
| 1.2285714285714286 | 0.027023491805306745 | 0.813953488372093 | 0.6957671957671958 | 28 | 3 | FALSE | Dyspnea | 20.821428571428573 | 0 | 28 | 0 | 0.9923809523809524 | TRUE | 0 | Dyspnea | 274 | 0.6123949579831933 |
| 1.3142857142857143 | 0.007943967221580478 | 0.7608695652173914 | 0.81 | 25 | 3 | FALSE | Abdominal stomach pain | 22.44 | 0 | 25 | 0 | 0.9895238095238096 | TRUE | 0 | Abdominal stomach pain | 122 | 0.66 |
| 1.6 | 0.0028251300520208086 | 0.625 | 0.9 | 16 | 3 | FALSE | Diarrhea | 24.6875 | 0 | 16 | 0 | 0.98 | TRUE | 0 | Diarrhea | 24 | 0.7481060606060606 |
| 1.2285714285714286 | 0.034130502441729146 | 0.813953488372093 | 0.698005698005698 | 27 | 2 | FALSE | Insomnia | 21 | 0 | 27 | 0 | 0.9923809523809524 | TRUE | 0 | Insomnia | 300 | 0.6 |
| 1.8 | 0.0016810663659403153 | 0.5555555555555556 | 0.75 | 8 | 3 | FALSE | Anxiety | 20.5 | 0 | 8 | 0 | 0.9733333333333333 | TRUE | 0 | Anxiety | 20 | 0.6029411764705882 |
| 1.8857142857142857 | 0 | 0.5303030303030303 | 1 | 5 | 3 | FALSE | Cognitive dysfunction | 26.2 | 0 | 5 | 0 | 0.9704761904761905 | TRUE | 0 | Cognitive dysfunction | 0 | 0.7705882352941177 |
| 1.3714285714285714 | 0.03144117488207414 | 0.7291666666666666 | 0.7944664031620553 | 23 | 3 | FALSE | Nausea Vomit | 22.304347826086957 | 0 | 23 | 0 | 0.9876190476190476 | TRUE | 0 | Nausea Vomit | 248 | 0.6560102301790282 |
| 1.4857142857142858 | 0.007930656252195394 | 0.673076923076923 | 0.8128654970760234 | 19 | 3 | FALSE | Fever | 23.105263157894736 | 0 | 19 | 0 | 0.9838095238095238 | TRUE | 0 | Fever | 76 | 0.6795665634674922 |
| 1.3428571428571427 | 0.010344997563787291 | 0.7446808510638299 | 0.841897233201581 | 23 | 2 | FALSE | Attention deficit | 23.217391304347824 | 0 | 23 | 0 | 0.9885714285714287 | TRUE | 0 | Attention deficit | 122 | 0.6633540372670808 |
| 1.7142857142857142 | 0.05875622976463312 | 0.5833333333333334 | 0.6727272727272727 | 11 | 3 | FALSE | Weakness in the limbs | 20.818181818181817 | 0 | 11 | 0 | 0.9761904761904762 | TRUE | 0 | Weakness in the limbs | 354 | 0.628099173553719 |
| 1.6857142857142857 | 1.984126984126984E-4 | 0.5932203389830508 | 0.9743589743589743 | 13 | 3 | FALSE | Tinnitus | 25.846153846153847 | 0 | 13 | 0 | 0.9771428571428571 | TRUE | 0 | Tinnitus | 4 | 0.7832167832167832 |
| 1.6857142857142857 | 0 | 0.5932203389830508 | 1 | 12 | 3 | FALSE | Expectoration | 25.916666666666668 | 0 | 12 | 0 | 0.9771428571428571 | TRUE | 0 | Expectoration | 0 | 0.7622549019607843 |
| 1.6 | 1.7740429505135386E-4 | 0.625 | 0.9809523809523809 | 15 | 3 | FALSE | Weight loss | 25.666666666666668 | 0 | 15 | 0 | 0.98 | TRUE | 0 | Weight loss | 4 | 0.7549019607843137 |
| 1.9714285714285715 | 0 | 0.5072463768115941 | 1 | 5 | 3 | FALSE | Chest tightness | 26.6 | 0 | 5 | 0 | 0.9676190476190476 | TRUE | 0 | Chest tightness | 0 | 0.8580645161290322 |
| 1.3142857142857143 | 0.012230784914657226 | 0.7608695652173914 | 0.7833333333333333 | 25 | 3 | FALSE | Paresthesia | 22.08 | 0 | 25 | 0 | 0.9895238095238096 | TRUE | 0 | Paresthesia | 150 | 0.6494117647058824 |
| 1.9142857142857144 | 0 | 0.5223880597014925 | 1 | 7 | 3 | FALSE | Seeing problems | 26 | 0 | 7 | 0 | 0.9695238095238096 | TRUE | 0 | Seeing problems | 0 | 0.8387096774193549 |
| 2.257142857142857 | 0 | 0.44303797468354433 | 1 | 2 | 4 | FALSE | Edema | 23 | 0 | 2 | 0 | 0.9580952380952381 | TRUE | 0 | Edema | 0 | 0.92 |
| 1.6571428571428573 | 0 | 0.603448275862069 | 1 | 13 | 3 | FALSE | Rhinorrhea | 25.692307692307693 | 0 | 13 | 0 | 0.978095238095238 | TRUE | 0 | Rhinorrhea | 0 | 0.755656108597285 |
| 2.085714285714286 | 0 | 0.4794520547945205 | 1 | 2 | 3 | FALSE | Constipation | 27.5 | 0 | 2 | 0 | 0.9638095238095238 | TRUE | 0 | Constipation | 0 | 0.9166666666666666 |
| 2.6857142857142855 | 0 | 0.37234042553191493 | 0 | 1 | 4 | FALSE | Swallowing problems | 11 | 0 | 1 | 0 | 0.9438095238095238 | TRUE | 0 | Swallowing problems | 0 | 0 |

**Table S4B 6^th^ Month Follow-Up**

| AverageShortestPathLength | BetweennessCentrality | ClosenessCentrality | ClusteringCoefficient | Degree | Eccentricity | IsSingleNode | name | NeighborhoodConnectivity | NumberOfDirectedEdges | NumberOfUndirectedEdges | PartnerOfMultiEdgedNodePairs | Radiality | selected | SelfLoops | shared name | Stress | TopologicalCoefficient |
| --- | --- | --- | --- | --- | --- | --- | --- | --- | --- | --- | --- | --- | --- | --- | --- | --- | --- |
| 2.466666666666667 | 0 | 0.4054054054054054 | 1 | 2 | 3 | FALSE | Abdominal pain | 13 | 0 | 2 | 0 | 0.9435897435897436 | FALSE | 0 | Abdominal pain | 0 | 0.9285714285714286 |
| 1.5333333333333334 | 0.03930440549608909 | 0.6521739130434783 | 0.8131868131868132 | 14 | 2 | FALSE | Diarrhea | 19.071428571428573 | 0 | 14 | 0 | 0.9794871794871794 | FALSE | 0 | Diarrhea | 248 | 0.6357142857142857 |
| 1.6333333333333333 | 0.02644028179018036 | 0.6122448979591837 | 0.8484848484848485 | 12 | 3 | FALSE | Sore throat | 18.833333333333332 | 0 | 12 | 0 | 0.9756410256410256 | FALSE | 0 | Sore throat | 186 | 0.6494252873563219 |
| 1.3333333333333333 | 0.01699015946747344 | 0.75 | 0.7666666666666667 | 21 | 3 | FALSE | Anxiety | 19.238095238095237 | 0 | 21 | 0 | 0.9871794871794872 | FALSE | 0 | Anxiety | 110 | 0.6633825944170771 |
| 1.7333333333333334 | 0 | 0.5769230769230769 | 1 | 10 | 3 | FALSE | Arrhythmia | 23.2 | 0 | 10 | 0 | 0.9717948717948718 | FALSE | 0 | Arrhythmia | 0 | 0.8285714285714286 |
| 1.5 | 0.0017532658911969257 | 0.6666666666666666 | 0.925 | 16 | 3 | FALSE | Attention deficit | 21.4375 | 0 | 16 | 0 | 0.9807692307692307 | FALSE | 0 | Attention deficit | 20 | 0.7392241379310345 |
| 1.3 | 0.05179725622743664 | 0.7692307692307692 | 0.7285714285714285 | 21 | 2 | FALSE | Chest pain | 19.095238095238095 | 0 | 21 | 0 | 0.9884615384615384 | FALSE | 0 | Chest pain | 250 | 0.6365079365079365 |
| 1.6333333333333333 | 5.069260241674034E-4 | 0.6122448979591837 | 0.9615384615384616 | 13 | 3 | FALSE | Cognitive dysfunction | 21.846153846153847 | 0 | 13 | 0 | 0.9756410256410256 | FALSE | 0 | Cognitive dysfunction | 6 | 0.7802197802197802 |
| 1.3 | 0.01932270559312302 | 0.7692307692307692 | 0.7272727272727273 | 22 | 3 | FALSE | Cough | 18.636363636363637 | 0 | 22 | 0 | 0.9884615384615384 | FALSE | 0 | Cough | 138 | 0.6426332288401254 |
| 1.2666666666666666 | 0.01766020011723012 | 0.7894736842105263 | 0.7351778656126482 | 23 | 3 | FALSE | Depression | 18.869565217391305 | 0 | 23 | 0 | 0.9897435897435898 | FALSE | 0 | Depression | 146 | 0.6506746626686657 |
| 1.3666666666666667 | 0.019460721833946985 | 0.7317073170731707 | 0.7602339181286549 | 19 | 2 | FALSE | Dizziness | 19.157894736842106 | 0 | 19 | 0 | 0.9858974358974358 | FALSE | 0 | Dizziness | 118 | 0.6385964912280702 |
| 1.2666666666666666 | 0.032274163557125014 | 0.7894736842105263 | 0.696969696969697 | 22 | 2 | FALSE | Dyspnea | 18.545454545454547 | 0 | 22 | 0 | 0.9897435897435898 | FALSE | 0 | Dyspnea | 172 | 0.6181818181818182 |
| 1.1333333333333333 | 0.06208318003716743 | 0.8823529411764706 | 0.6276923076923077 | 26 | 2 | FALSE | Fatigue | 17.5 | 0 | 26 | 0 | 0.9948717948717949 | FALSE | 0 | Fatigue | 318 | 0.5833333333333334 |
| 1.2666666666666666 | 0.02900670811421319 | 0.7894736842105263 | 0.70995670995671 | 22 | 2 | FALSE | Hair loss | 18.681818181818183 | 0 | 22 | 0 | 0.9897435897435898 | FALSE | 0 | Hair loss | 178 | 0.6227272727272727 |
| 1.4333333333333333 | 0.018875007495697153 | 0.6976744186046512 | 0.7189542483660131 | 18 | 3 | FALSE | Headache | 18.61111111111111 | 0 | 18 | 0 | 0.9833333333333333 | FALSE | 0 | Headache | 98 | 0.6417624521072797 |
| 1.2 | 0.026336053600385833 | 0.8333333333333334 | 0.7101449275362319 | 24 | 2 | FALSE | Insomnia | 18.625 | 0 | 24 | 0 | 0.9923076923076923 | FALSE | 0 | Insomnia | 220 | 0.6208333333333333 |
| 1.2 | 0.02683997496637616 | 0.8333333333333334 | 0.7065217391304348 | 24 | 2 | FALSE | Joint pain | 18.583333333333332 | 0 | 24 | 0 | 0.9923076923076923 | FALSE | 0 | Joint pain | 218 | 0.6194444444444445 |
| 1.2333333333333334 | 0.021215708027387325 | 0.8108108108108107 | 0.7351778656126482 | 23 | 2 | FALSE | Loss of Smell | 18.956521739130434 | 0 | 23 | 0 | 0.991025641025641 | FALSE | 0 | Loss of Smell | 182 | 0.6318840579710145 |
| 1.2 | 0.024772636174899443 | 0.8333333333333334 | 0.7210144927536232 | 24 | 2 | FALSE | Loss of Taste | 18.75 | 0 | 24 | 0 | 0.9923076923076923 | FALSE | 0 | Loss of Taste | 214 | 0.625 |
| 1.2333333333333334 | 0.043664066658622 | 0.8108108108108107 | 0.6557971014492754 | 24 | 3 | FALSE | Memory deficit | 17.791666666666668 | 0 | 24 | 0 | 0.991025641025641 | FALSE | 0 | Memory deficit | 210 | 0.6135057471264368 |
| 1.3333333333333333 | 0.023396837081047606 | 0.75 | 0.7238095238095238 | 21 | 3 | FALSE | Myalgia | 18.571428571428573 | 0 | 21 | 0 | 0.9871794871794872 | FALSE | 0 | Myalgia | 132 | 0.6403940886699507 |
| 1.6333333333333333 | 7.624942107700728E-4 | 0.6122448979591837 | 0.9393939393939394 | 12 | 3 | FALSE | Nausea Vomit | 21.583333333333332 | 0 | 12 | 0 | 0.9756410256410256 | FALSE | 0 | Nausea Vomit | 8 | 0.7442528735632183 |
| 1.3333333333333333 | 0.010875892569604535 | 0.75 | 0.8105263157894737 | 20 | 2 | FALSE | Palpitations | 19.95 | 0 | 20 | 0 | 0.9871794871794872 | FALSE | 0 | Palpitations | 106 | 0.665 |
| 1.9 | 3.448275862068965E-4 | 0.5263157894736842 | 0.8 | 5 | 3 | FALSE | Paresthesia | 20.8 | 0 | 5 | 0 | 0.9653846153846154 | FALSE | 0 | Paresthesia | 4 | 0.7428571428571429 |
| 1.5333333333333334 | 0.02396314237778954 | 0.6521739130434783 | 0.8285714285714286 | 15 | 3 | FALSE | Weakness in the limbs | 20.466666666666665 | 0 | 15 | 0 | 0.9794871794871794 | FALSE | 0 | Weakness in the limbs | 124 | 0.7057471264367816 |
| 1.7333333333333334 | 1.532567049808429E-4 | 0.5769230769230769 | 0.9722222222222222 | 9 | 3 | FALSE | Seeing problems | 22.444444444444443 | 0 | 9 | 0 | 0.9717948717948718 | FALSE | 0 | Seeing problems | 2 | 0.7739463601532567 |
| 1.4666666666666666 | 0.006397272249199226 | 0.6818181818181819 | 0.8416666666666667 | 16 | 2 | FALSE | Skin rash | 20.125 | 0 | 16 | 0 | 0.9820512820512821 | FALSE | 0 | Skin rash | 58 | 0.6708333333333333 |
| 2.2 | 0 | 0.45454545454545453 | 1 | 2 | 3 | FALSE | Sweating | 18 | 0 | 2 | 0 | 0.9538461538461539 | FALSE | 0 | Sweating | 0 | 0.8181818181818182 |
| 1.9333333333333333 | 0 | 0.5172413793103449 | 1 | 4 | 3 | FALSE | Chest tightness | 23.25 | 0 | 4 | 0 | 0.9641025641025641 | FALSE | 0 | Chest tightness | 0 | 0.8303571428571429 |
| 1.8666666666666667 | 0 | 0.5357142857142857 | 1 | 6 | 3 | FALSE | Weight loss | 22.166666666666668 | 0 | 6 | 0 | 0.9666666666666667 | FALSE | 0 | Weight loss | 0 | 0.7916666666666666 |
| 1.6333333333333333 | 6.304423545802858E-4 | 0.6122448979591837 | 0.9696969696969697 | 12 | 3 | FALSE | Decreased appetite | 20.583333333333332 | 0 | 12 | 0 | 0.9756410256410256 | FALSE | 0 | Decreased appetite | 8 | 0.7097701149425287 |

**Table S4C 12^th^ Month Follow-Up**

| AverageShortestPathLength | BetweennessCentrality | ClosenessCentrality | ClusteringCoefficient | Degree | Eccentricity | IsSingleNode | name | NeighborhoodConnectivity | NumberOfDirectedEdges | NumberOfUndirectedEdges | PartnerOfMultiEdgedNodePairs | Radiality | selected | SelfLoops | shared name | Stress | TopologicalCoefficient |
| --- | --- | --- | --- | --- | --- | --- | --- | --- | --- | --- | --- | --- | --- | --- | --- | --- | --- |
| 1.4242424242424243 | 0.008278332291490187 | 0.7021276595744681 | 0.7485380116959064 | 19 | 2 | FALSE | Anxiety | 21.94736842105263 | 0 | 19 | 0 | 0.9853709508881923 | FALSE | 0 | Anxiety | 92 | 0.6650717703349283 |
| 1.303030303030303 | 0.010090400601720263 | 0.7674418604651163 | 0.7865612648221344 | 23 | 2 | FALSE | Chest pain | 22.130434782608695 | 0 | 23 | 0 | 0.9895506792058516 | FALSE | 0 | Chest pain | 112 | 0.6706192358366272 |
| 1.696969696969697 | 0.002070018050131687 | 0.5892857142857143 | 0.7818181818181819 | 11 | 3 | FALSE | Chest tightness | 22.181818181818183 | 0 | 11 | 0 | 0.9759665621734588 | FALSE | 0 | Chest tightness | 24 | 0.6931818181818182 |
| 1.606060606060606 | 0.001798603663005225 | 0.6226415094339623 | 0.8571428571428571 | 14 | 3 | FALSE | Cognitive dysfunction | 23.5 | 0 | 14 | 0 | 0.9791013584117032 | FALSE | 0 | Cognitive dysfunction | 26 | 0.734375 |
| 1.121212121212121 | 0.04439055076610488 | 0.891891891891892 | 0.6477832512315271 | 29 | 2 | FALSE | Cough | 20.137931034482758 | 0 | 29 | 0 | 0.9958202716823407 | FALSE | 0 | Cough | 292 | 0.6102403343782654 |
| 1.4242424242424243 | 0.005402472653739189 | 0.7021276595744681 | 0.8105263157894737 | 20 | 3 | FALSE | Depression | 22.6 | 0 | 20 | 0 | 0.9853709508881923 | FALSE | 0 | Depression | 72 | 0.70625 |
| 1.2424242424242424 | 0.019124558897506052 | 0.8048780487804879 | 0.7033333333333334 | 25 | 2 | FALSE | Diarrhea | 21.04 | 0 | 25 | 0 | 0.9916405433646813 | FALSE | 0 | Diarrhea | 182 | 0.6375757575757576 |
| 1.1818181818181819 | 0.07592326496179756 | 0.8461538461538461 | 0.6780626780626781 | 27 | 2 | FALSE | Dizziness | 20.666666666666668 | 0 | 27 | 0 | 0.9937304075235109 | FALSE | 0 | Dizziness | 336 | 0.6446759259259259 |
| 1.1818181818181819 | 0.030100390169126096 | 0.8461538461538461 | 0.7094017094017094 | 27 | 2 | FALSE | Dyspnea | 21.074074074074073 | 0 | 27 | 0 | 0.9937304075235109 | FALSE | 0 | Dyspnea | 210 | 0.6386083052749719 |
| 1.5454545454545454 | 0.0016160210940590197 | 0.6470588235294118 | 0.8857142857142857 | 15 | 2 | FALSE | Edema | 23.8 | 0 | 15 | 0 | 0.9811912225705328 | FALSE | 0 | Edema | 24 | 0.7212121212121212 |
| 1.5151515151515151 | 0.005633027783763078 | 0.66 | 0.7583333333333333 | 16 | 2 | FALSE | Expectoration | 22.25 | 0 | 16 | 0 | 0.9822361546499477 | FALSE | 0 | Expectoration | 60 | 0.6742424242424242 |
| 1.1515151515151516 | 0.06658799953440929 | 0.8684210526315789 | 0.58994708994709 | 28 | 2 | FALSE | Fatigue | 19.214285714285715 | 0 | 28 | 0 | 0.9947753396029257 | FALSE | 0 | Fatigue | 322 | 0.5822510822510822 |
| 1.393939393939394 | 0.003276514648726512 | 0.717391304347826 | 0.8736842105263158 | 20 | 2 | FALSE | Hair loss | 23.85 | 0 | 20 | 0 | 0.9864158829676071 | FALSE | 0 | Hair loss | 50 | 0.7227272727272728 |
| 1.2424242424242424 | 0.019442144186418396 | 0.8048780487804879 | 0.72 | 25 | 2 | FALSE | Insomnia | 21.24 | 0 | 25 | 0 | 0.9916405433646813 | FALSE | 0 | Insomnia | 174 | 0.6436363636363637 |
| 1.1818181818181819 | 0.017439965576883317 | 0.8461538461538461 | 0.7264957264957265 | 27 | 2 | FALSE | Joint pain | 21.296296296296298 | 0 | 27 | 0 | 0.9937304075235109 | FALSE | 0 | Joint pain | 198 | 0.6453423120089786 |
| 1.2727272727272727 | 0.03184248090159468 | 0.7857142857142857 | 0.6630434782608695 | 24 | 2 | FALSE | Loss of Taste | 20.458333333333332 | 0 | 24 | 0 | 0.9905956112852664 | FALSE | 0 | Loss of Taste | 194 | 0.6199494949494949 |
| 1.1818181818181819 | 0.018012798678125514 | 0.8461538461538461 | 0.7236467236467237 | 27 | 2 | FALSE | Myalgia | 21.296296296296298 | 0 | 27 | 0 | 0.9937304075235109 | FALSE | 0 | Myalgia | 200 | 0.6453423120089786 |
| 1.2121212121212122 | 0.014844024060487262 | 0.825 | 0.7446153846153846 | 26 | 2 | FALSE | Palpitations | 21.653846153846153 | 0 | 26 | 0 | 0.9926854754440961 | FALSE | 0 | Palpitations | 172 | 0.6561771561771562 |
| 1.3333333333333333 | 0.004449184262238372 | 0.75 | 0.8614718614718615 | 22 | 2 | FALSE | Paresthesia | 23.318181818181817 | 0 | 22 | 0 | 0.9885057471264368 | FALSE | 0 | Paresthesia | 68 | 0.7066115702479339 |
| 1.6666666666666667 | 0.0015953165100892374 | 0.6 | 0.8363636363636363 | 11 | 2 | FALSE | Sweating | 23.272727272727273 | 0 | 11 | 0 | 0.9770114942528735 | FALSE | 0 | Sweating | 20 | 0.7052341597796143 |
| 1.4545454545454546 | 0.001491735102416217 | 0.6875 | 0.9215686274509803 | 19 | 2 | FALSE | Arrhythmia | 24.38888888888889 | 0 | 19 | 1 | 0.9843260188087775 | FALSE | 0 | Arrhythmia | 26 | 0.7390572390572391 |
| 1.4242424242424243 | 0.003297112654521886 | 0.7021276595744681 | 0.8771929824561403 | 19 | 2 | FALSE | Attention deficit | 23.42105263157895 | 0 | 19 | 0 | 0.9853709508881923 | FALSE | 0 | Attention deficit | 46 | 0.7097288676236044 |
| 1.3636363636363635 | 0.005705378922554549 | 0.7333333333333334 | 0.819047619047619 | 22 | 2 | FALSE | Decreased appetite | 22.523809523809526 | 0 | 22 | 1 | 0.987460815047022 | FALSE | 0 | Decreased appetite | 76 | 0.6825396825396826 |
| 1.2424242424242424 | 0.011931630051001004 | 0.8048780487804879 | 0.76 | 25 | 2 | FALSE | Headache | 21.8 | 0 | 25 | 0 | 0.9916405433646813 | FALSE | 0 | Headache | 148 | 0.6606060606060606 |
| 1.1818181818181819 | 0.0392047725230071 | 0.8461538461538461 | 0.6581196581196581 | 27 | 2 | FALSE | Loss of Smell | 20.37037037037037 | 0 | 27 | 0 | 0.9937304075235109 | FALSE | 0 | Loss of Smell | 248 | 0.6172839506172839 |
| 1.2727272727272727 | 0.012361436706620354 | 0.7857142857142857 | 0.7536231884057971 | 24 | 2 | FALSE | Memory deficit | 21.875 | 0 | 24 | 0 | 0.9905956112852664 | FALSE | 0 | Memory deficit | 140 | 0.6628787878787878 |
| 1.4545454545454546 | 0.004454247042816561 | 0.6875 | 0.8431372549019608 | 18 | 2 | FALSE | Skin rash | 22.944444444444443 | 0 | 18 | 0 | 0.9843260188087775 | FALSE | 0 | Skin rash | 50 | 0.6952861952861953 |
| 1.606060606060606 | 4.1045126184445074E-4 | 0.6226415094339623 | 0.9615384615384616 | 13 | 2 | FALSE | Weakness in the limbs | 24.615384615384617 | 0 | 13 | 0 | 0.9791013584117032 | FALSE | 0 | Weakness in the limbs | 8 | 0.745920745920746 |
| 2.1515151515151514 | 0 | 0.46478873239436624 | 0 | 1 | 3 | FALSE | Chills | 27 | 0 | 1 | 0 | 0.9602925809822361 | FALSE | 0 | Chills | 0 | 0 |
| 1.5757575757575757 | 0.0033405880071549925 | 0.6346153846153847 | 0.7692307692307693 | 14 | 2 | FALSE | Sore throat | 23 | 0 | 14 | 0 | 0.9801462904911181 | FALSE | 0 | Sore throat | 46 | 0.696969696969697 |
| 1.8484848484848484 | 1.8576917500362478E-4 | 0.5409836065573771 | 0.8666666666666667 | 6 | 3 | FALSE | Hearing problems | 26.333333333333332 | 0 | 6 | 0 | 0.9707419017763845 | FALSE | 0 | Hearing problems | 4 | 0.8229166666666666 |
| 1.5757575757575757 | 0.0016079001707343952 | 0.6346153846153847 | 0.8791208791208791 | 14 | 2 | FALSE | Nausea Vomit | 23.714285714285715 | 0 | 14 | 0 | 0.9801462904911181 | FALSE | 0 | Nausea Vomit | 22 | 0.7186147186147186 |
| 1.9393939393939394 | 0 | 0.515625 | 1 | 3 | 3 | FALSE | Rhinorrhea | 28 | 0 | 3 | 0 | 0.9676071055381401 | FALSE | 0 | Rhinorrhea | 0 | 0.875 |
| 1.9393939393939394 | 0 | 0.515625 | 1 | 3 | 3 | FALSE | Fever | 26.333333333333332 | 0 | 3 | 0 | 0.9676071055381401 | FALSE | 0 | Fever | 0 | 0.8229166666666666 |

**Table S4D 24^th^ Month Follow-Up**

| AverageShortestPathLength | BetweennessCentrality | ClosenessCentrality | ClusteringCoefficient | Degree | Eccentricity | IsSingleNode | name | NeighborhoodConnectivity | NumberOfDirectedEdges | NumberOfUndirectedEdges | PartnerOfMultiEdgedNodePairs | Radiality | selected | SelfLoops | shared name | Stress | TopologicalCoefficient |
| --- | --- | --- | --- | --- | --- | --- | --- | --- | --- | --- | --- | --- | --- | --- | --- | --- | --- |
| 2.5 | 0 | 0.4 | 1 | 2 | 4 | FALSE | Anxiety | 8 | 0 | 2 | 0 | 0.90625 | FALSE | 0 | Anxiety | 0 | 0.6666666666666666 |
| 1.7916666666666667 | 0.12430124223602487 | 0.5581395348837209 | 0.42857142857142855 | 8 | 3 | FALSE | Fatigue | 8.75 | 0 | 8 | 0 | 0.9505208333333334 | FALSE | 0 | Fatigue | 262 | 0.43125 |
| 1.6666666666666667 | 0.013284653116350171 | 0.6 | 0.8214285714285714 | 8 | 2 | FALSE | Hair loss | 12.5 | 0 | 8 | 0 | 0.9583333333333334 | FALSE | 0 | Hair loss | 50 | 0.5208333333333334 |
| 1.3333333333333333 | 0.18927226414954323 | 0.75 | 0.4166666666666667 | 16 | 2 | FALSE | Dizziness | 9.4375 | 0 | 16 | 0 | 0.9791666666666666 | FALSE | 0 | Dizziness | 346 | 0.4076086956521739 |
| 1.5833333333333333 | 0.03309860526725884 | 0.6315789473684211 | 0.6363636363636364 | 12 | 3 | FALSE | Diarrhea | 11 | 0 | 12 | 0 | 0.9635416666666666 | FALSE | 0 | Diarrhea | 94 | 0.5 |
| 1.5 | 0.10785789420957724 | 0.6666666666666666 | 0.5604395604395604 | 14 | 3 | FALSE | Loss of Smell | 10.214285714285714 | 0 | 14 | 0 | 0.96875 | FALSE | 0 | Loss of Smell | 190 | 0.48299319727891155 |
| 1.5833333333333333 | 0.016690737772434825 | 0.6315789473684211 | 0.7121212121212122 | 12 | 3 | FALSE | Loss of Taste | 11.583333333333334 | 0 | 12 | 0 | 0.9635416666666666 | FALSE | 0 | Loss of Taste | 74 | 0.5265151515151515 |
| 1.5833333333333333 | 0.019214261596800168 | 0.6315789473684211 | 0.696969696969697 | 12 | 3 | FALSE | Decreased appetite | 11.416666666666666 | 0 | 12 | 0 | 0.9635416666666666 | FALSE | 0 | Decreased appetite | 82 | 0.5189393939393939 |
| 1.8333333333333333 | 0.017236024844720498 | 0.5454545454545455 | 0.6111111111111112 | 9 | 4 | FALSE | Headache | 10.777777777777779 | 0 | 9 | 0 | 0.9479166666666666 | FALSE | 0 | Headache | 46 | 0.5388888888888889 |
| 1.5 | 0.04630214014856371 | 0.6666666666666666 | 0.6515151515151515 | 12 | 2 | FALSE | Palpitations | 11.083333333333334 | 0 | 12 | 0 | 0.96875 | FALSE | 0 | Palpitations | 132 | 0.4618055555555556 |
| 1.4583333333333333 | 0.11729052108610313 | 0.6857142857142857 | 0.5238095238095238 | 15 | 3 | FALSE | Sore throat | 10.066666666666666 | 0 | 15 | 0 | 0.9713541666666666 | FALSE | 0 | Sore throat | 224 | 0.47619047619047616 |
| 2.0416666666666665 | 0.0018909036265277502 | 0.489795918367347 | 0.6666666666666666 | 4 | 3 | FALSE | Cough | 12.25 | 0 | 4 | 0 | 0.9348958333333334 | FALSE | 0 | Cough | 14 | 0.6447368421052632 |
| 1.75 | 0.058340754098117335 | 0.5714285714285714 | 0.42857142857142855 | 8 | 3 | FALSE | Memory deficit | 8.25 | 0 | 8 | 0 | 0.953125 | FALSE | 0 | Memory deficit | 114 | 0.375 |
| 1.7083333333333333 | 0.028047105159657755 | 0.5853658536585367 | 0.4888888888888889 | 10 | 3 | FALSE | Dyspnea | 10.1 | 0 | 10 | 0 | 0.9557291666666666 | FALSE | 0 | Dyspnea | 82 | 0.48095238095238096 |
| 1.4166666666666667 | 0.06058006263125478 | 0.7058823529411764 | 0.5934065934065934 | 14 | 2 | FALSE | Nausea Vomit | 10.928571428571429 | 0 | 14 | 0 | 0.9739583333333334 | FALSE | 0 | Nausea Vomit | 176 | 0.45535714285714285 |
| 1.7083333333333333 | 0.02238636026328873 | 0.5853658536585367 | 0.6111111111111112 | 9 | 3 | FALSE | Myalgia | 10.666666666666666 | 0 | 9 | 0 | 0.9557291666666666 | FALSE | 0 | Myalgia | 64 | 0.48484848484848486 |
| 1.9583333333333333 | 0.00322061191626409 | 0.5106382978723405 | 0.7 | 5 | 3 | FALSE | Attention deficit | 11 | 0 | 5 | 0 | 0.9401041666666666 | FALSE | 0 | Attention deficit | 10 | 0.55 |
| 1.9166666666666667 | 0.0027173913043478256 | 0.5217391304347826 | 0.8095238095238095 | 7 | 3 | FALSE | Skin rash | 11.857142857142858 | 0 | 7 | 0 | 0.9427083333333334 | FALSE | 0 | Skin rash | 8 | 0.6240601503759399 |
| 1.7083333333333333 | 0.03021874559392091 | 0.5853658536585367 | 0.5 | 9 | 3 | FALSE | Insomnia | 10.222222222222221 | 0 | 9 | 0 | 0.9557291666666666 | FALSE | 0 | Insomnia | 84 | 0.46464646464646464 |
| 1.7083333333333333 | 0.002577556267948975 | 0.5853658536585367 | 0.9166666666666666 | 9 | 3 | FALSE | Chills | 12.88888888888889 | 0 | 9 | 0 | 0.9557291666666666 | FALSE | 0 | Chills | 20 | 0.5858585858585859 |
| 1.75 | 0.014892454566367608 | 0.5714285714285714 | 0.6190476190476191 | 7 | 3 | FALSE | Joint pain | 11.285714285714286 | 0 | 7 | 0 | 0.953125 | FALSE | 0 | Joint pain | 46 | 0.4906832298136646 |
| 2.75 | 0 | 0.36363636363636365 | 0 | 1 | 4 | FALSE | Chest tightness | 8 | 0 | 1 | 0 | 0.890625 | FALSE | 0 | Chest tightness | 0 | 0 |
| 2.4583333333333335 | 0 | 0.4067796610169491 | 0 | 1 | 4 | FALSE | Chest pain | 14 | 0 | 1 | 0 | 0.9088541666666666 | FALSE | 0 | Chest pain | 0 | 0 |
| 2.4166666666666665 | 0 | 0.4137931034482759 | 0 | 1 | 4 | FALSE | Paresthesia | 15 | 0 | 1 | 0 | 0.9114583333333334 | FALSE | 0 | Paresthesia | 0 | 0 |
| 2.2916666666666665 | 0 | 0.4363636363636364 | 0 | 1 | 3 | FALSE | Depression | 16 | 0 | 1 | 0 | 0.9192708333333334 | FALSE | 0 | Depression | 0 | 0 |

**Figure S1** **Forest Plot of Symptom Prevalence at Different Follow-Up Periods**

**Figure S1A 3^rd^ Follow-up**

**
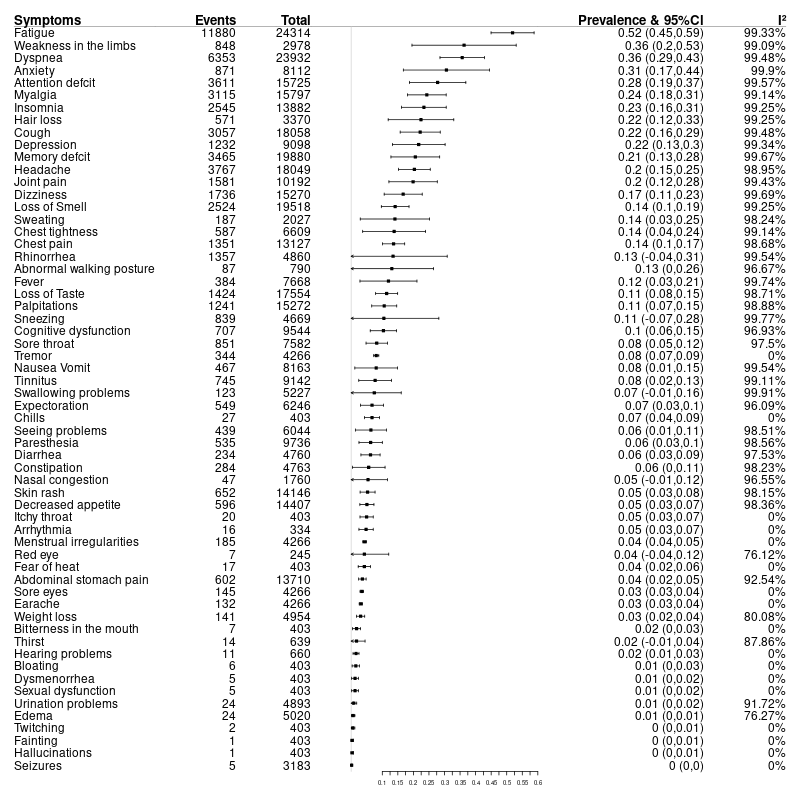
**

**Figure S1B 6^th^ Follow-up**

**
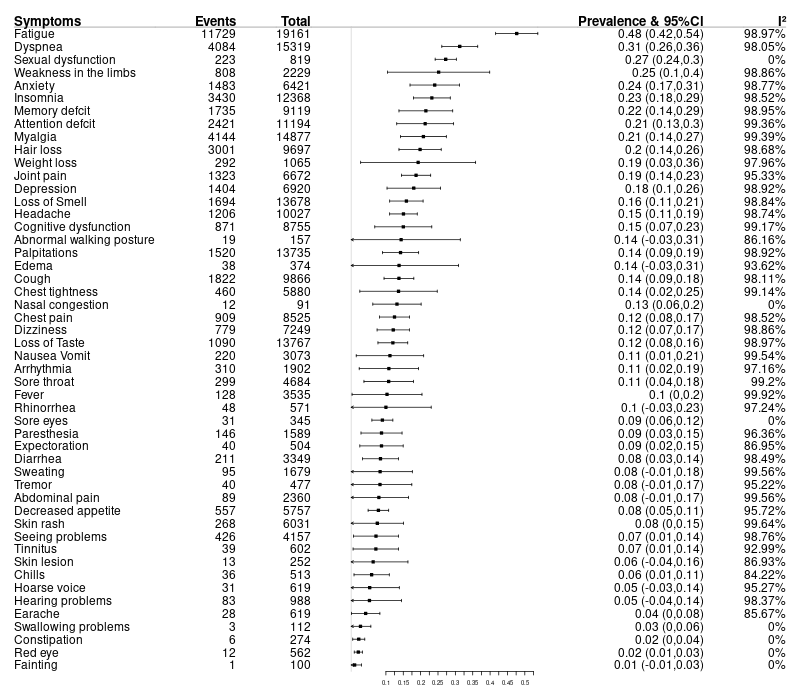
**

**Figure S1C 12^th^ Follow-up**

**
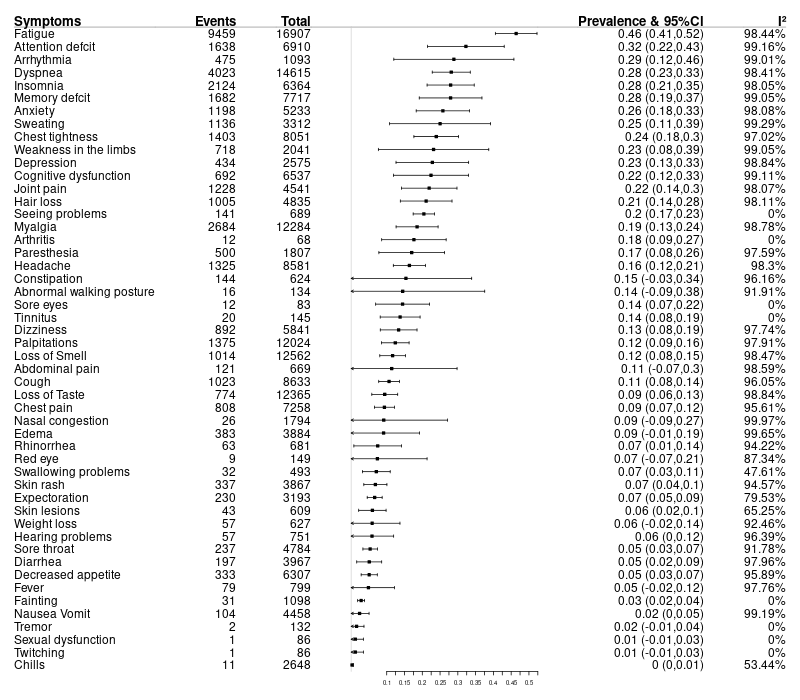
**

**Figure S1D 24^th^ Follow-up**

**
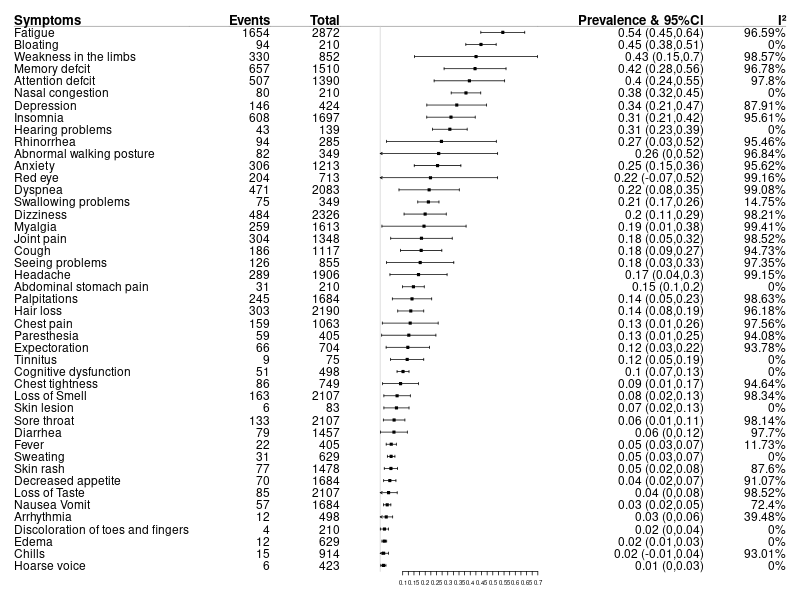
**

**Figure S1E All Follow-up Periods**

**
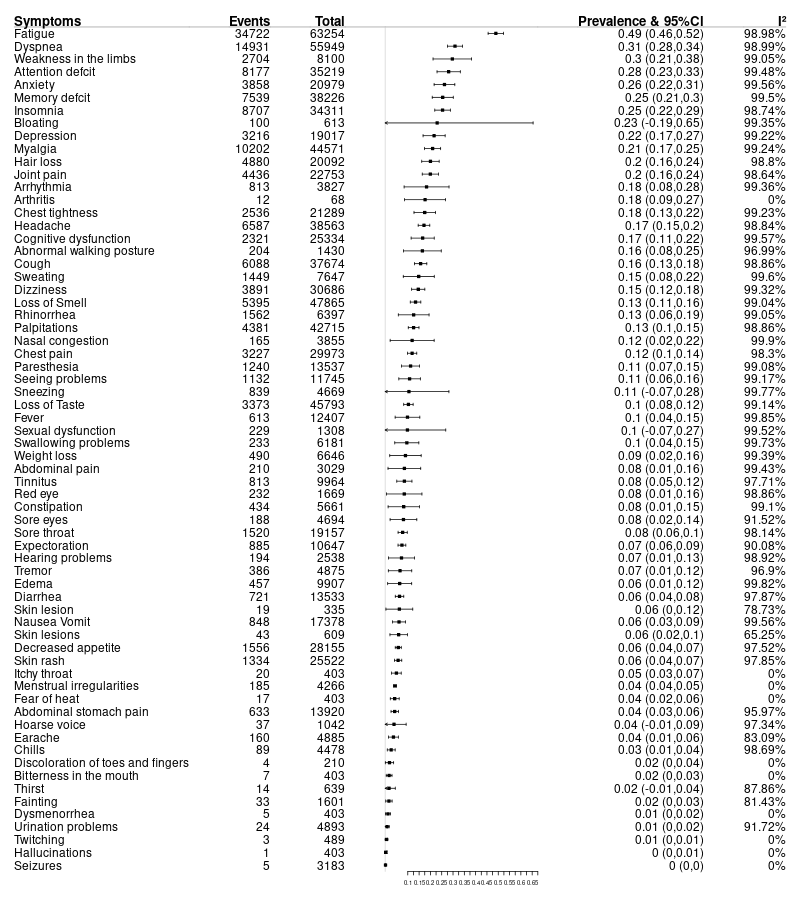
**

**Figure S2 Clustering analysis of clinical symptoms by severity at the other three intervals**

**
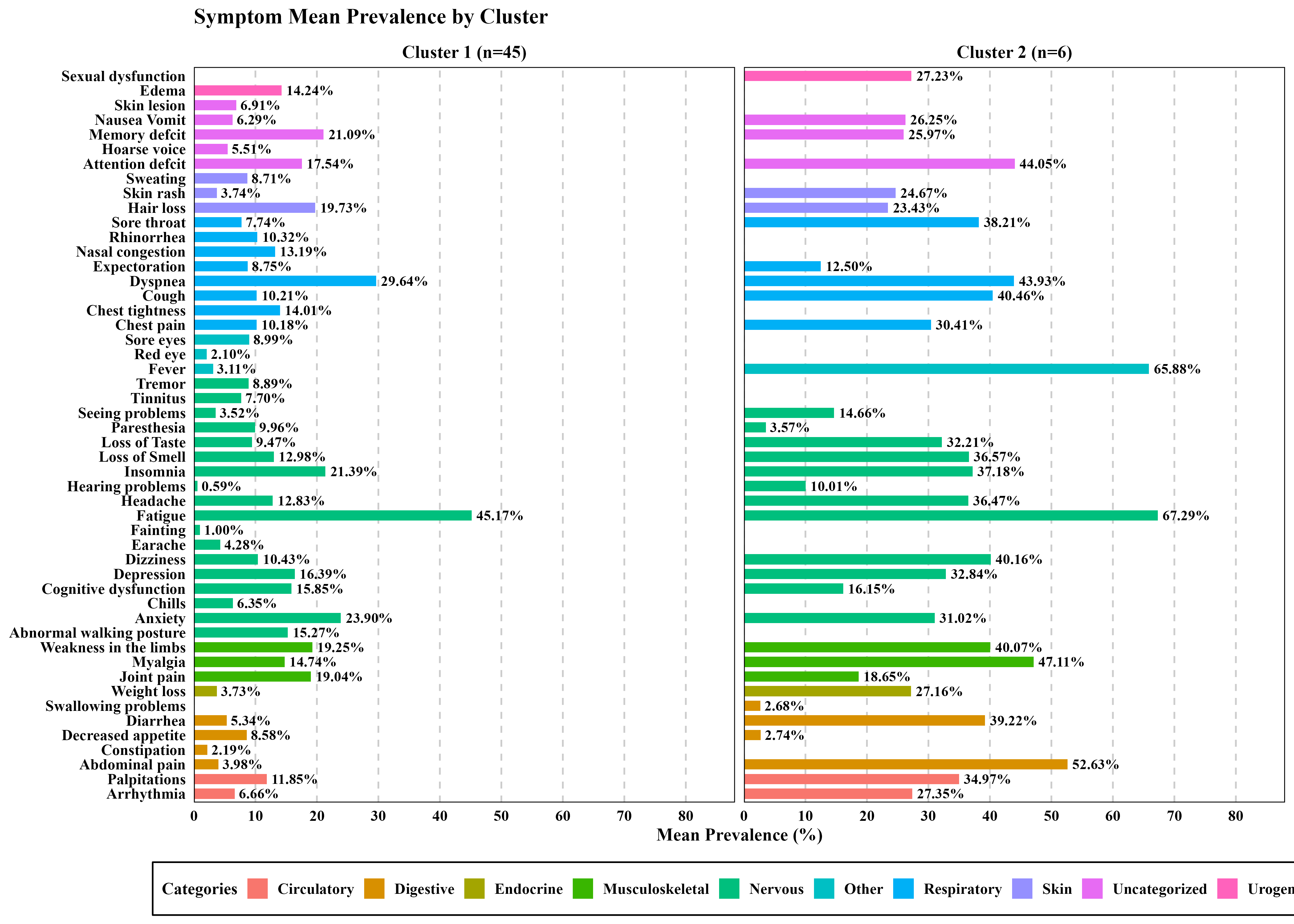
**

**The 6^th^ Follow-up**

**
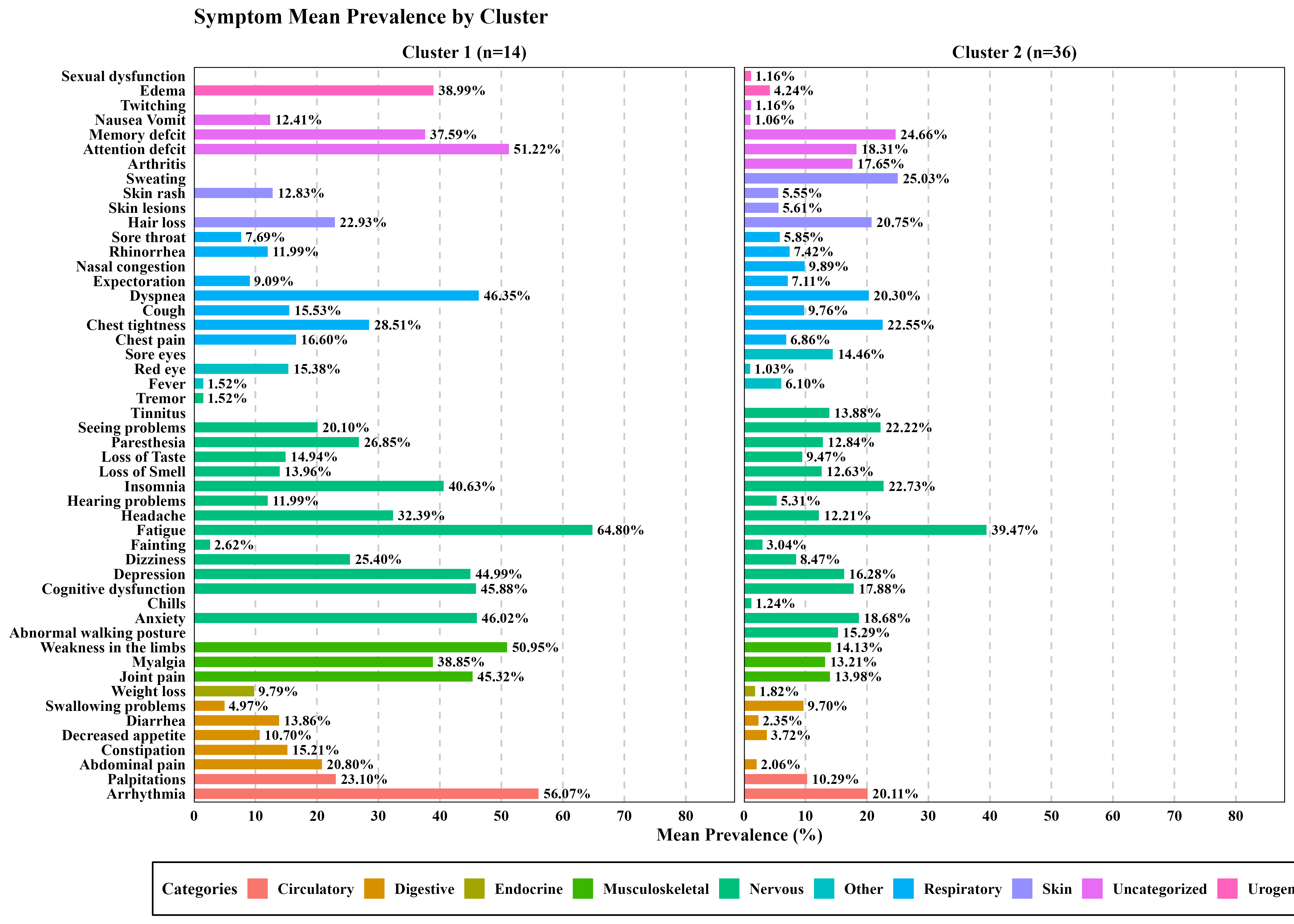
**

**The 12^th^ Follow-up**
