# Supplementary material for: Symptom Trajectories and Clinical Subtypes in Post–COVID-19 Condition: Systematic Review and Clustering Analysis
Source: JMIR Public Health Surveill. 2025 Jul 18;11:e72221. doi: 10.2196/72221 (PMC12296217; doi:10.2196/72221)
Supplement: Multimedia Appendix 2 [file publichealth-v11-e72221-s002.doc]

**Search strategy**

**PubMed**

**#1**

("observational study"[All Fields] OR "observational research"[All Fields] OR "cohort study"[All Fields] OR "case-control study"[All Fields] OR "cross-sectional study"[All Fields] OR "longitudinal study"[All Fields] OR "retrospective study"[All Fields] OR "prospective study"[All Fields])

**#2**

("symptoms"[Title/Abstract] OR "characteristics"[Title/Abstract] OR "clinical characteristics"[Title/Abstract] OR "clinical symptoms"[Title/Abstract] OR "cases"[Title/Abstract] OR "cases report"[Title/Abstract])

**#3**

(("Post-Acute COVID-19 Syndrome"[MeSH Terms] OR ("covid 19 syndrome post acute"[Title/Abstract] OR "Post-Acute COVID-19 Syndrome"[Title/Abstract] OR "post acute covid 19 syndromes"[Title/Abstract] OR "long haul covid"[Title/Abstract] OR "covid long haul"[Title/Abstract] OR "long haul covid"[Title/Abstract] OR ("Long-Haul"[All Fields] AND "COVIDs"[Title/Abstract]) OR "long haul covid 19"[Title/Abstract] OR "covid 19 long haul"[Title/Abstract] OR "long haul covid 19"[Title/Abstract] OR (("Long"[All Fields] AND "Haul"[All Fields]) AND "COVID-19s"[Title/Abstract]) OR "post acute sequelae of covid 19"[Title/Abstract] OR "covid 19 post acute sequelae"[Title/Abstract] OR "post acute sequelae of covid 19"[Title/Abstract] OR "post acute sequelae of sars cov 2 infection"[Title/Abstract] OR "post acute sequelae of sars cov 2 infection"[Title/Abstract] OR "post covid conditions"[Title/Abstract] OR "post covid conditions"[Title/Abstract] OR "post covid condition"[Title/Abstract] OR "long covid"[Title/Abstract] OR "pasc post acute sequelae of covid 19"[Title/Abstract] OR "pasc post acute sequelae of covid 19"[Title/Abstract] OR "Post-Acute COVID-19 Syndrome"[Title/Abstract]))

**#4**

("humans"[MeSH Terms] AND "english"[Language])

**#5**

("review"[Publication Type])

**#6**

(#1 AND #2 AND #3 AND #4) NOT #5

**Embase**

**#1**

'long covid'/exp OR 'long covid' OR 'chronic covid syndrome':ab,ti OR 'chronic covid-19':ab,ti OR 'covid long-hauler':ab,ti OR 'covid-19 long-hauler':ab,ti OR 'long haul covid':ab,ti OR 'long haul covid-19':ab,ti OR 'long hauler covid':ab,ti OR 'post covid 19 fatigue':ab,ti OR 'post covid 19 neurological syndrome':ab,ti OR 'post covid 19 syndrome':ab,ti OR 'post covid fatigue':ab,ti OR 'post covid impairment':ab,ti OR 'post covid syndrome':ab,ti OR 'post-acute covid syndrome':ab,ti OR 'post-acute covid-19':ab,ti OR 'post-acute covid-19 fatigue':ab,ti OR 'post-acute covid-19 neurological syndrome':ab,ti OR 'post-acute covid-19 syndrome':ab,ti OR 'post-acute sequelae of sars-cov-2 infection':ab,ti OR 'post-covid condition':ab,ti OR 'post-covid-19 condition':ab,ti OR 'long covid':ab,ti

**#2**

'observational study':ab,ti OR 'observational research':ab,ti OR 'cohort study':ab,ti OR 'case-control study':ab,ti OR 'cross-sectional study':ab,ti OR 'longitudinal study':ab,ti OR 'retrospective study':ab,ti OR 'prospective study':ab,ti

**#3**

'symptoms':ab,ti OR 'characteristics':ab,ti OR 'clinical characteristics':ab,ti OR 'clinical symptoms':ab,ti OR 'cases':ab,ti OR 'cases report':ab,ti

**#4**

#1 AND #2 AND #3

**#5**

#1 AND #2 AND #3 AND [article]/lim AND [humans]/lim AND [english]/lim AND [clinical study]/lim
