# Supplementary figures and images for: Symptom Trajectories and Clinical Subtypes in Post–COVID-19 Condition: Systematic Review and Clustering Analysis
Source: JMIR Public Health Surveill. 2025 Jul 18;11:e72221. doi: 10.2196/72221 (PMC12296217; doi:10.2196/72221)

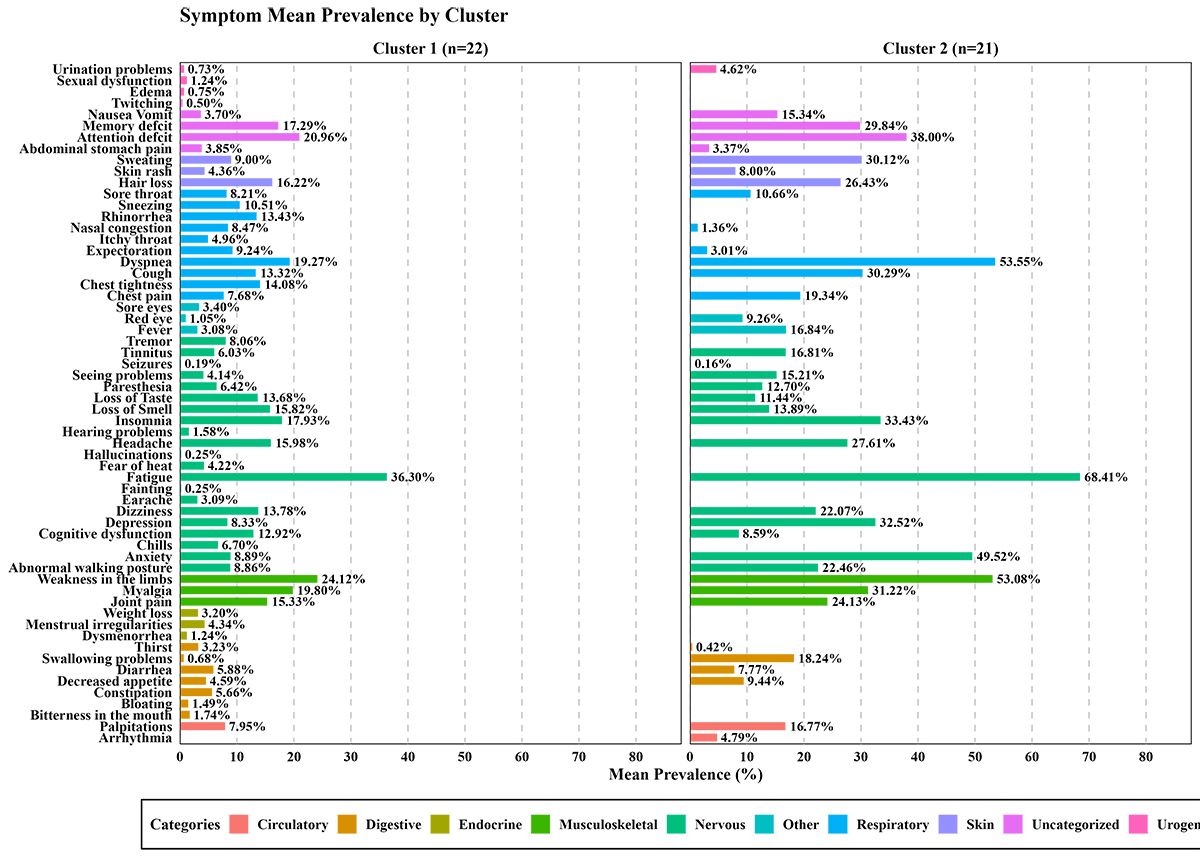

Supplement: Multimedia Appendix 3 [file publichealth-v11-e72221-s003.png]
